# Ironing out the question: what is limiting cyanobacteria in freshwater lakes in the Prairie Pothole Region?

**DOI:** 10.1007/s10533-025-01234-7

**Published:** 2025-05-20

**Authors:** Irena F. Creed, Owen Salmon, Kevin J. Erratt, Charles G. Trick

**Affiliations:** Department of Physical & Environmental Sciences, University of Toronto, Toronto, ON Canada

**Keywords:** Phytoplankton, Nitrogen, Phosphorus, Iron, Nutrient limitation, Siderophores

## Abstract

The Canadian Prairie Pothole Region is a notable hotspot for cyanobacteria-dominated lakes. This study found minor variations in cyanobacterial genera across these lakes yet observed significant differences in standing biomass, as the lakes ranged from oligotrophic to hypereutrophic classifications. A correlational analysis of nutrients, specifically total phosphorus (TP) and total nitrogen (TN) revealed that the limiting nutrients varied considerably across the region. Of the lakes studied, cyanobacterial biomass was P-limited in 21 lakes, N-limited in 3 lakes, and co-limited by both P and N in 23 lakes. Surprisingly, in 32 lakes, the biomass was limited by neither P nor N. In these lakes, iron (Fe) emerged as the most likely limiting nutrient, given a relatively narrow range of free ferric Fe (pFe) between 18 and 26. Cyanobacteria can create biomass under Fe stress by producing Fe-scavenging siderophores that target pFe. However, in neither P- nor N-limited lakes, there was a lack of correlation between siderophore concentrations and cyanobacterial biomass (r = 0.05), indicating that the siderophores were unable to scavenge Fe and thereby utilize the available P and N to produce further cyanobacterial biomass. Our findings suggest that these Fe-starved eutrophic lakes exhibited a paradox of slow-growing yet high cyanobacterial biomass, challenging the notion that only oligotrophic lakes embody slow-growing metabolisms. Overall, our study highlights the importance of considering nutrient limitations on cyanobacterial growth and incorporating macro- (P and N) and micro- (Fe) nutrient limitation considerations into existing nutrient management strategies to mitigate cyanobacterial dominance effectively.

**Supplementary Information:**

The online version contains supplementary material available at 10.1007/s10533-025-01234-7.

## Introduction

Reports of increasing cyanobacterial dominance in lakes (Brooks et al. 2016; Visser et al. 2016; Wells et al. 2020) have raised concerns due to the adverse impacts of cyanobacteria on lake food webs and the health and well-being of human communities reliant on these water sources (Schindler and Vallentyne 2008). Cyanobacterial dominance, often mistakenly referred to as cyanobacterial blooms, occurs when more than 50% of the phytoplankton assemblage is dominated by cyanobacteria, regardless of whether surface “scum” accumulation is observed (Molot et al. 2014; Erratt et al. 2022). With climate change expected to escalate temperatures and increase the frequency and magnitude of extreme weather events, there is a heightened risk that cyanobacteria will proliferate in lakes, even those where they would not typically thrive (Paul 2008; Reichwaldt and Ghadouani 2012; Erratt et al. 2023). Without a deeper understanding of the environmental factors influencing cyanobacteria, our capacity to recommend effective interventions for mitigating cyanobacteria dominance remains limited.

In freshwaters, the two nutrients most closely associated with cyanobacterial dominance are phosphorus (P) and nitrogen (N), and there is a substantial debate about the importance of each, even after more than 50 years of study (Conley et al. 2009). These macronutrients can alter the trophic state of lakes, shifting from nutrient-poor conditions (i.e., oligotrophic) characterized by low phytoplankton productivity to nutrient-rich conditions (i.e., eutrophic) associated with high phytoplankton productivity often linked with cyanobacterial dominance (Carlson 1977). Different scientific perspectives have emerged to predict cyanobacterial dominance in freshwater lakes (Schindler 1977; Paerl and Millie 1996). The first perspective, introduced by Schindler (1977), asserts that P is the limiting nutrient for cyanobacteria in freshwater systems. Schindler’s research demonstrated that reducing P concentrations in lakes effectively limits cyanobacterial growth and prevents cyanobacterial dominance, even if N is in low concentrations, as  select cyanobacteria can fix atmospheric N to satisfy their N needs (Schindler 1977; Schindler et al. 2016). According to Schindler, P is the essential nutrient to target for controlling cyanobacterial dominance (Schindler 1977). The second perspective, introduced by Paerl et al. (2016, 2020), asserts that both P and N are nutrients that can limit cyanobacterial growth, depending on their availability. According to Paerl et al. (2016), reducing only one of these nutrients—typically P, according to Schindler (1977)—may not control cyanobacterial dominance, as cyanobacteria may shift their reliance to the next most important nutrient. Instead, both P and N should be reduced. Xu et al. (2015) proposed specific critical thresholds for P and N for cyanobacterial dominance. In their study of Lake Taihu, a shallow eutrophic lake in China, they identified 0.05 mg L^−1^ total P (TP) and 0.8 mg L^−1^ total N (TN) as the minimum predictive concentrations for cyanobacterial dominance. Below these thresholds, cyanobacterial growth is constrained by the limiting nutrient. These perspectives have shaped our understanding of the nutrient requirements of prokaryotic cyanobacteria, which are often more problematic than competing eukaryotic algae (Reynolds 2006).

Efforts to relate P, N, and the ratio of N:P to cyanobacterial dominance in freshwater lakes have shown varying degrees of success (e.g., Downing et al. 2001). These mixed results may stem from the influence of other, often overlooked, limiting factors, such as the availability of trace metals that may be critical in regulating cyanobacterial growth. Molot et al. (2014) argued that the supply of micronutrients—notably iron (Fe), but also molybdenum and copper—should be considered, given the capacity of these to modulate the assimilation and metabolism of macronutrients for cyanobacteria. Thus, if cyanobacteria can obtain or use micronutrients more efficiently than eukaryotic species, then the pool of macronutrients would preferentially be transferred to cyanobacteria. There is a growing need to expand beyond traditional perspectives and incorporate additional variables into management strategies. Integrating factors such as trace metal availability into our models can enhance our understanding and improve our capacity to effectively manage and mitigate cyanobacterial dominance in aquatic ecosystems.

**Fig. 1:**
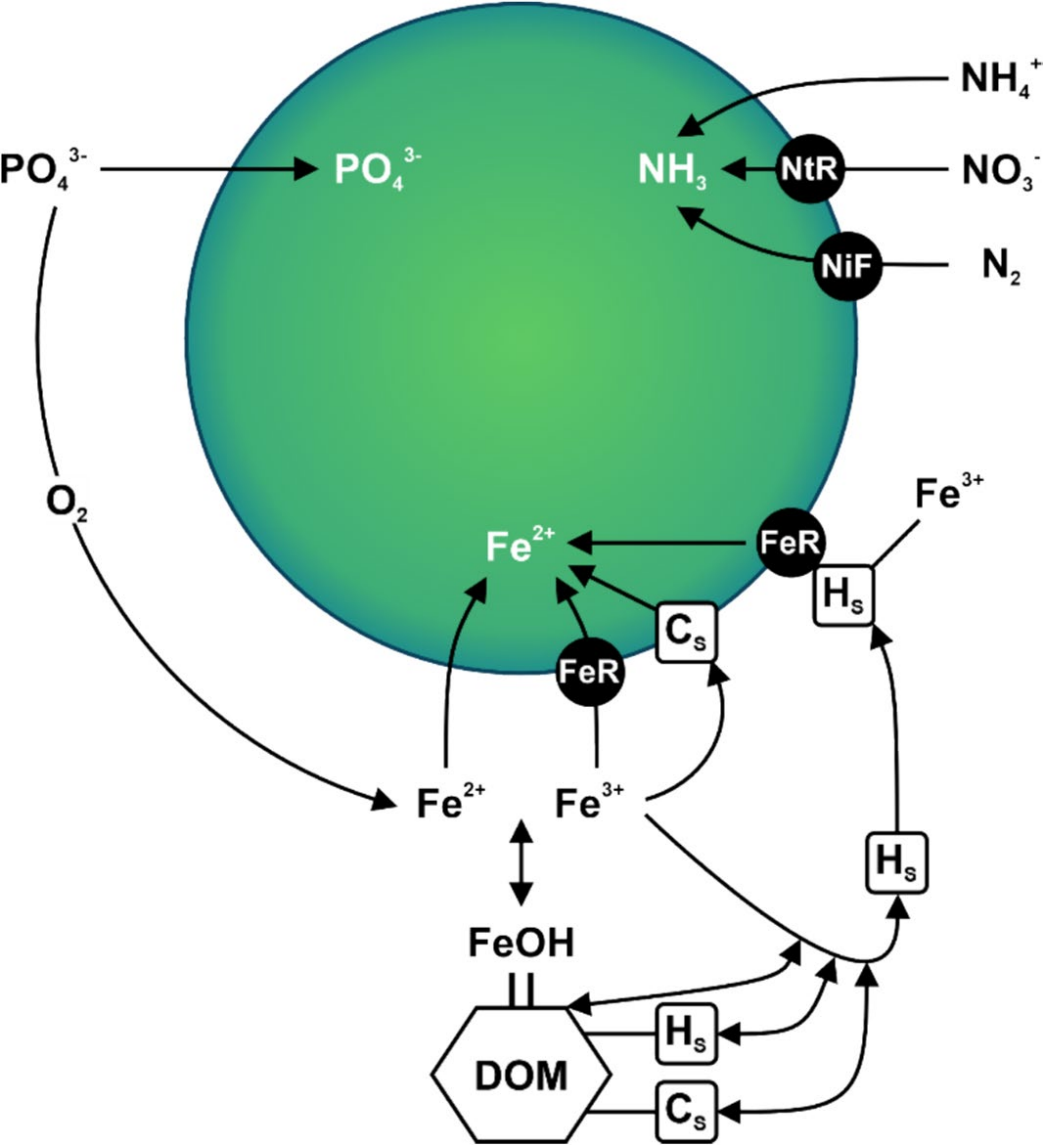
Enhanced Fe transport through hydroxamate and catecholate siderophore production corresponds to the metabolic needs of macronutrient acquisition. In the hydroxamate siderophore (HS) system, there is a low ferric iron (Fe^3+^) binding efficiency, in which the HS is released to the external environment and re-absorbed by random chance after the cyanobacteria bind and reduce Fe^3+^ to ferrous iron (Fe^2+^) (Årstøl and Hohmann-Marriott 2019). In contrast, the catecholate Fe transport system (CS) has a high Fe^3+^ binding efficiency, where catecholate Fe attaches to the cell wall and absorbs Fe^3+^ that encounters the cyanobacteria cell (Wilhelm and Trick 1994). DOM = dissolved organic matter; FeOH = ferric oxyhydroxide; N_2_ = nitrogen molecule; NO_3_^−^ = nitrate-nitrogen; NH_4_^+^  = ammonium; NH_3_ = ammonia; O_2_ = oxygen molecule; PO_4_^3−^ = phosphate; NiF = nitrogenase; NtR = nitrate reductase; FeR = ferric reductase (modified after Sorichetti et al. 2014a)

Fe may become the limiting nutrient for cyanobacteria when both N and P are plentiful. Cyanobacteria require Fe for various critical functions, such as photosynthesis and N uptake [i.e., nitrate (NO_3_^−^) assimilation and atmospheric N gas (N_2_) fixation] (Wilhelm 1995) (Fig. 1), and cyanobacteria need approximately ten times more Fe than eukaryotic algae (Gonzalez et al. 2018). However, the high demand for Fe challenges cyanobacteria, as ferric iron (Fe^3^⁺), the commonly occurring oxidative state under oxic conditions, readily forms biologically unavailable precipitates (Shaked and Lis 2012). This results in Fe concentrations of approximately pFe 20 to pFe 8, where pFe refers to the negative logarithm (log_10_) of Fe^3^⁺ concentration in water, with a higher pFe indicating lower ferric Fe concentration (Wilhelm and Trick 1994; Sunda and Huntsman 2015; Gonzalez et al. 2018; Ghio and Hillborn 2024).

Bioavailable ferrous Fe (Fe^2^⁺) is quickly oxidized and transformed into insoluble Fe^3^⁺ (Shaked and Lis 2012; Xiao et al. 2021, 2022). Fe starvation, occurring above pFe16, inhibits cyanobacterial growth and reduces their competitive ability to dominate over eukaryotic algae (Wilhelm and Trick 1994). Studies have shown that N_2_-fixing cyanobacteria genera have a higher Fe demand than non-N_2_-fixing genera (e.g., Hyenstrand et al. 2000; Xiao et al. 2021). In these two studies, despite other essential nutrients being available, Fe availability was crucial in the growth, competition, and survival of N_2_-fixing cyanobacteria.

In response to Fe-limitation, cyanobacteria can secrete Fe-binding siderophores to scavenge pFe and outcompete eukaryotic algae for a limited Fe pool (Murphy et al. 1976; Wilhelm and Trick 1994; Sorichetti et al. 2014a). Alternatively, cyanobacteria can use siderophores secreted by other microorganisms, e.g., fungi and bacteria, which decompose organic matter in the contributing catchment, contributing to both dissolved organic matter (DOM) and siderophores in runoff to lakes (Barbeau et al. 2003; Sorichetti et al. 2014b). Cyanobacteria can use siderophores to cleave Fe from Fe-DOM complexes, especially DOM with labile properties, due to its low Fe binding efficiency (Sorichetti et al. 2014b). Siderophores have a high affinity for Fe^3+^ and form soluble Fe^3+^ complexes, facilitating active transport and subsequent reduction to Fe^2+^ into the cyanobacterial cell (Murphy et al. 1976; Wilhelm and Trick 1994; Wilhelm et al. 1998). Two main types of siderophores are used by cyanobacteria: hydroxamate siderophores are excreted to the external environment and have a weak Fe-binding efficiency, and catecholate siderophores are bound to the cell membrane and have a high Fe-binding efficiency (Sorichetti et al. 2014a). Cyanobacteria studied across lakes ranging from oligotrophic to hypereutrophic have all shown the ability to produce siderophores to access Fe in limiting environments (Sorichetti et al. 2014a, b; Sorichetti et al. 2016; Du et al. 2019). Despite the different nutrient concentrations in lakes, siderophores were observed when Fe was the limiting nutrient, and cyanobacteria was the dominant phytoplankton group in these studies.

This study aims to establish the relationships between lake chemistry and cyanobacterial biomass in lakes within the Prairie Pothole Region of western Canada. A single time-point sampling approach was applied during the peak cyanobacterial bloom season (July to August) in the Canadian Prairies (MacKeigan et al. 2023). This snapshot approach was selected to capture nutrient limitations when cyanobacteria are most metabolically active and when resource competition is likely most pronounced (Reynolds 2006). A temporally intensive design would provide insights into seasonal dynamics. However, the spatially extensive design used here enables the identification of broad-scale patterns of nutrient limitations and cyanobacterial metabolism across a wide geographic gradient. This approach is particularly useful for assessing mechanistic drivers of cyanobacterial growth limitation as it allows comparisons across lakes with varying nutrient availability, land use, and natural and human influences.

The study aims to assess the connection between nutrient levels and peak cyanobacterial biomass to determine whether understanding the availability of P, N, or Fe can predict cyanobacteria community dynamics. Through a large-scale lake survey and systematic correlational assessments, this study tests the following hypotheses:

### Hypothesis 1 (H1)

Cyanobacteria in all lakes are P or N-limited. We predict that cyanobacteria in lakes will be macronutrient-limited, regardless of other environmental parameters.

### Hypothesis 2 (H2)

P or N do not limit cyanobacteria in lakes in agriculturally dominated landscapes. We predict that cyanobacteria in lakes with substantial human impact within the contributing catchment that drains into the lake will be neither P- nor N-limited but Fe-limited.

### Hypothesis 3 (H3)

Cyanobacteria in lakes that are neither P- nor N-limited cannot benefit from added P or N due to the lack of Fe. We predict that cyanobacteria can scavenge Fe by producing and using siderophores to maintain the cyanobacteria community.

## Methods

### Study area

The Canadian Prairie Pothole Region (PPR) spans over 550,000 km^2^ across southern and central Alberta, Saskatchewan, and southern Manitoba. The climate of the PPR is classified as semi-arid to sub-humid, with significant seasonal and annual variability in precipitation (Winter 1989; Conly and Van der Kamp 2001). Annual mean temperatures range from + 1 to + 6 °C, and annual total precipitation ranges from 350 to 650 mm, which is often less than the annual mean evapotranspiration of 600 to 800 mm (Whitfield et al. 2024; Uchytil 2024). Due to this imbalance between water gains and losses during the summer, a significant portion of groundwater recharge comes from spring snowmelt.

The PPR is characterized by a distinctive landscape of glacially formed depressions known as potholes. These water-filled depressions create numerous small, shallow lakes crucial to the region’s hydrology and ecology (Millar 1976). Often hydrologically isolated, potholes capture water from precipitation, playing a vital role in groundwater recharge and nutrient retention, which helps prevent overland runoff to downstream waters (Whitfield et al. 2024). Many lakes in the PPR are polymictic, characterized by an unstable thermal regime due to shallow bathymetry and strong wind forcing in the region. These physical factors promote frequent mixing events, preventing the establishment of stable thermal stratification and contributing to highly dynamic thermal conditions throughout the open-water season (Orihel et al. 2015). As a result, bottom waters in these lakes often maintain oxygen levels above the anoxia threshold of 0.5 mg L⁻^1^ dissolved oxygen (Wetzel 2001). However, these systems may experience episodic periods of anoxia—rather than the prolonged anoxic conditions observed in dimictic systems—particularly during calm conditions or at night when biological oxygen demand exceeds oxygen replenishment (Wetzel 2001). These cycles of oxygen depletion and reoxygenation create highly variable redox conditions that influence nutrient availability and microbial community dynamics.

The lakes of the PPR hold significant recreational value (Nanayakkara and Wissel 2017). These lakes support an active angling community, with many waterbodies experiencing heavy fishing pressure leading to declines in fishery stocks and the subsequent implementation of active recovery programs (Sullivan et al. 2003; Blackwell et al. 2019). Additionally, the lakes are used for boating and swimming (Gordy et al. 2018), and many boat access points are located in provincial parks. The shallow nature of these lakes, coupled with accelerated climate warming and agricultural intensification, creates favourable conditions for the proliferation of cyanobacteria, particularly during the warmer months (Erratt et al. 2023). There is evidence that the risk of seasonal exposure to potentially harmful cyanobacteria has already risen and will continue to increase under future climate projections, especially as a society actively engages with these waters during periods of peak cyanobacteria activity (e.g., warmer summer months) (Hayes et al. 2020).

Agricultural activities cover over 38% of the land area in the Canadian portion of the PPR (Government of Canada 2020). Consequently, lakes in the PPR are highly susceptible to nutrient enrichment due to the region’s agricultural practices. The cultivation of crops, particularly canola and wheat, combined with livestock farming, has led to increased P and N loading into surface water bodies, accelerating the process of eutrophication (Howard et al. 2006). The shallow nature of these lakes, coupled with accelerated climate warming and agricultural intensification, creates favourable conditions for the proliferation of cyanobacteria, particularly during the warmer months (Erratt et al. 2023). The frequency and intensity of cyanobacterial dominance are expected to increase with ongoing climate change (Winter et al. 2011), posing significant public health risks due to their potential to produce harmful toxins such as microcystins (MC) (Paerl and Otten 2013).

### Lake selection

**Fig. 2:**
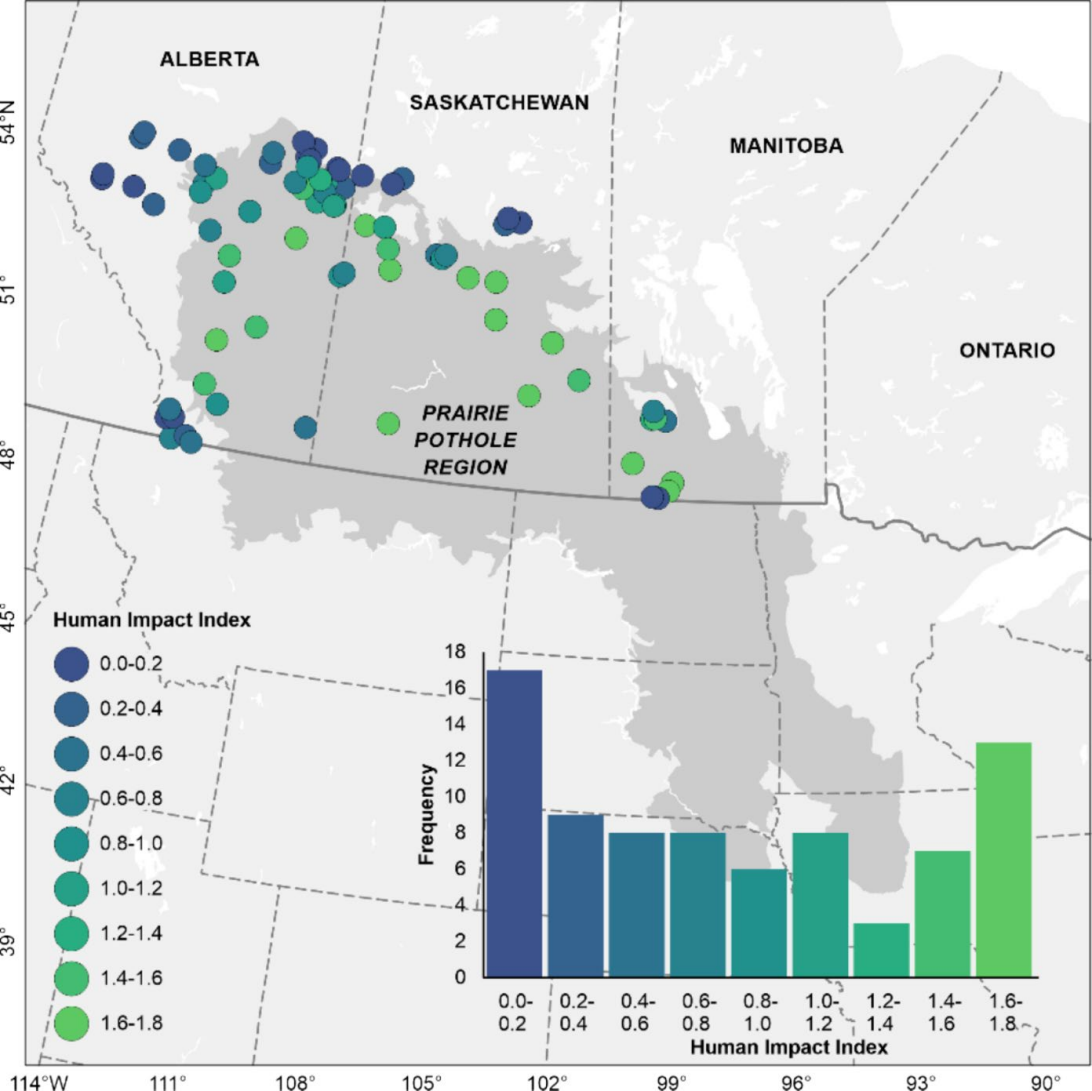
Locations and mean Human Impact Index values (ranging from 0 to 2) within the contributing catchments of the study lakes

A total of 79 lakes across Alberta, Saskatchewan, and Manitoba were selected to represent the range of human impacts within their contributing catchments (Fig. 2). Lake boundaries were obtained for 69 lakes from bathymetry charts and for 10 lakes (where bathymetries were unavailable) from the HydroLAKES database (version 1.0) (Messager et al. 2016). Catchment boundaries for each lake were delineated using the Watershed tool in Whitebox 3.3.0, employing a hydrologically conditioned 3-arc-second resolution HydroSHEDS Digital Elevation Model (DEM) (version 1.1) (Lehner et al. 2008) resampled to 90-m resolution. Exceptions were made for eight lakes, where catchment boundaries were delineated using: a 5-m High Resolution Digital Elevation Model (HRDEM; https://open.canada.ca/data/en/dataset/957782bf-847c-4644-a757-e383c0057995) resampled to 10-m resolution; a 1-arc-second Shuttle Radar Topography Mission (SRTM; https://www.earthdata.nasa.gov/sensors/srtm) resampled to 30-m resolution; a 30-m Canadian Digital Elevation Model (CDEM; https://open.canada.ca/data/en/dataset/7f245e4d-76c2-4caa-951a-45d1d2051333); or an existing published catchment boundary (i.e., for Minnie Lake (Alberta, 54.28°N, 111.10°W), where a catchment boundary was available from the Alberta Lake Management Society 2023). Lake areas were subsequently subtracted from catchment areas to produce catchment land areas. See Supplementary Information Table S1 for lake names, lake identifications (IDs), geographic coordinates, and lake and catchment boundary data sources.

To assess human impacts within each lake catchment, a Human Impact Index map was generated using 2020 land cover data from Agriculture and Agri-Food Canada (AAFC), Mines, Energy, and Communication Networks in Canada (CanVec), and the University of Maryland’s Global Forest Change (GFC) data. First, AAFC 30-m land cover classes were reclassified into three categories: 0 (natural/undisturbed, including water, bare land, rock, wetlands, and forests), 1 (pasturelands and grasslands), and 2 (urban and croplands). CanVec vector data, representing anthropogenic impacts from mining and energy, were converted to a 30-m raster aligned with the AAFC data and assigned a value of 2. Similarly, GFC 30-m data were aligned and categorized as 0 (no forest loss) or 1 (forest loss). These reclassified maps were then combined by selecting the maximum value per pixel, producing the Human Impact Index map with values of 0 (low), 1 (moderate), and 2 (high) human modification. Subsequently, mean Human Impact Index values, along with the percentages of agricultural cover (cropland plus pastureland and grassland), cropland, pastureland and grassland, and forest cover, were calculated within each lake’s catchment area using the AAFC land cover maps. All mapping procedures were performed in ArcGIS Pro (version 3.2.2).

### Lake physical properties

Fourteen physical properties were extracted for each lake: (1) catchment land area (km^2^), (2) percent wetland area in catchment, (3) percent wetland area connected via drainage networks to lake in catchment, (4) percent riparian area in catchment, (5) ratio of catchment land area to lake area, (6) shoreline length (m), (7) shoreline development, (8) lake fetch (km), (9) lake area (ha), (10) lake volume (m^3^), (11) mean lake depth (m), (12) maximum lake depth (m), (13) percent littoral zone in lake, and (14) dynamic ratio (km m^−1^).

The *catchment land area* is the total land area of a lake’s contributing catchment in km^2^—see “Lake selection” section for details on catchment boundary delineation. *Percent wetland area in catchment* represents the total area of wetlands in 2020 as a percentage of total catchment land area—2020 wetlands were defined by identifying 30 m pixels with a minimum frequency of 5 year inundation occurrence in annual surface water maps between 2011 and 2020 inclusive (Olthof and Rainville 2022), and subsequently removing lakes (HydroLAKES database; Messager et al. 2016) and single (unconnected) remaining pixels. *Percent wetland area connected *via* drainage networks to lake in catchment* represents the total area of wetlands that are hydrologically connected to the lake—hydrologically connected wetlands were defined as wetlands in 2020 (defined above) that spatially intersected watercourse and canal features connected to lakes (https://open.canada.ca/data/en/dataset/a4b190fe-e090-4e6d-881e-b87956c07977) as a percentage of total catchment land area. *Percent riparian area in catchment* represents the vegetated area adjacent to a lake shoreline as a percentage of total catchment land area—riparian areas were defined as contiguous groups of 30 m pixels classified as forest or shrubland from 2020 land cover maps from Agriculture and Agri-Food Canada (AAFC) (Government of Canada 2020) within 90 m of lake boundaries that spatially intersected the lake boundaries. *Ratio of catchment land area to lake area* represents the relative terrestrial nutrient loading area available to contribute to the total lake area. *Shoreline length* is the length of the lake surface water perimeter, including any islands, in m. *Shoreline development index* represents the relative amount of a lake perimeter that can receive nutrients from terrestrial sources where opportunities for terrestrial-water interactions increase with increasing shoreline development values—shoreline development index is calculated as the ratio of shoreline length to the circumference of a circle with the same lake area. *Lake fetch* represents the longest straight-line distance of open water that wind can travel across a lake in km, and can be used to characterize the potential for sediment and nutrient mixing. *Lake area* is the total surface area of the lake in ha—see “Lake selection” section for details on lake boundary delineation. *Lake volume* is the total volume of water in a lake in m^3^—depth contours and points from bathymetry maps were used to generate bathymetric grids at the spatial resolution of the DEMs used to extract catchment boundaries, and volumes below zero depth were subsequently calculated. *Mean lake depth* and *maximum lake depth* in m were both calculated from the bathymetric grids described above. *Percent littoral zone in lake* represents the relative area of a lake covered by the critical shallow zone near the shoreline characterized by warm temperatures, greater light penetration, and rooted vegetation like cattails and bulrushes—littoral zones were defined as contiguous bathymetry grid pixels ≤ 2 m depth that spatially intersected the lake boundaries. *Dynamic ratio* represents the proportion of a lakebed that is susceptible to sediment suspension and accumulation processes, where smaller values indicate a trend to bowl shapes and larger values indicate a trend to dish shapes (Casas-Ruiz et al. 2021)—dynamic ratio is calculated as √lake area in km^2^/mean depth in m (Hakanson 2005). The physical properties described above were extracted using ArcGIS Pro (version 3.2.2) and are provided for each lake in the Supplementary Information Table S2.

### Lake water chemical analyses

For this study, lakes were selected based on their freshwater status, with conductivity (µS cm⁻^1^) as a proxy for freshwater status. Conductivity was measured using a YSI EXO-2 multiparameter sonde at a 1 m depth, and lakes with conductivity < 3000 µS cm^−1^ were categorized as freshwater (Wetzel 2001). Lake sampling occurred during the peak phytoplankton biomass period in the study region (July–August) (MacKeigan et al. 2023). Lake water samples were collected from the center of each lake using a 1 m depth-integrated sampler to obtain a composite sample from the top meter of the water column. These samples were then analyzed for water chemistry and plankton composition, including cyanobacteria species and zooplankton functional groups (i.e., filter feeders vs. grazers).

We evaluated TP and TN concentrations relative to established threshold values to assess nutrient limitation across lakes. Following the criteria proposed by Xu et al. (2015), lakes with TP concentrations below 0.05 mg L⁻^1^ were classified as P-limited, while those with TN concentrations below 0.8 mg L⁻^1^ were classified as N-limited. Lakes with both TP and TN concentrations below 0.05 mg L⁻^1^ and 0.8 mg L⁻^1^, respectively, were considered co-limited by P and N. In contrast, those exceeding these nutrient thresholds were classified as neither P- nor N-limited.

TP, TN, and molar N:P were measured on unfiltered lake water samples. TP (μg L^−1^) was measured using Flow Injection Analysis (Lachat QuikChem 8500 FIA automated ion analyzer), where TP was converted to PO_4_^3−^ in a sulfuric acid-persulfate medium to produce a colored product that is measured spectrophotometrically (Estela and Cerdà 2005). TN (μg L^−1^) was measured using a Shimadzu TOC-V CPH with a TMN unit, where TN is measured by chemiluminescence (Ward et al. 1980).

Water transparency was assessed by lowering a Secchi disk into the water column until it was no longer visible (M1), at which point the depth was recorded (M1). The disk was then gradually raised until it became visible again (M2). Secchi depth was calculated as the average of M1 and M2 (i.e., M1 + M2)/2). Color (True color units, TCU), dissolved organic carbon (DOC, mg L^−1^), specific ultraviolet absorbance at 254 nm (SUVA_254_, L mg C^−1^ m^−1^), and fluorescence index (FI) were measured on samples filtered through a pre-combusted GF/F glass fiber filter (0.45 μm pore size). Color was determined by measuring absorbance at 410 nm using a Shimadzu UVmini-1240 spectrophotometer, with absorbance values transformed into TCU through a calibration curve built with Hazen’s Cobalt-Platinate Standard (where 1 mg Pt L^−1^ equals 1 TCU) (Hongve and Åkesson 1996). DOC, defined as the organic carbon that passes through a 0.45 μm pore-size filter (Thurman 1985), was measured using the non-purgeable organic carbon method (Deflandre and Gangé 2001) and reported in units of mg L^−1^. SUVA254 values, a proxy for DOM aromaticity (McKnight et al. 2001), were calculated by normalizing absorbance values at Ab 254 nm (measured using a Duetta-Fluorescence and Absorbance Spectrometer) to DOC concentrations (McKnight et al. 2001). Higher SUVA254 values (< 3 L mg C^−1^ m^−1^) indicate a greater proportion of aromatic compounds in the DOM that are generally less biodegradable and more complicated to degrade, and lower SUVA254 values (< 3 L mg C^−1^ m^−1^) indicate a greater proportion of aliphatic compounds that are generally more labile in the DOM and more biodegradable (Weishaar et al. 2003). FI was used as a proxy for DOM origin (McKnight et al. 2001). FI values were calculated as the ratio of emission intensity at 470 nm to that of 520 nm at an excitation of 370 nm (measured using a Duetta-Fluorescence and Absorbance Spectrometer). Higher FI values (> 1.8) indicate DOM of autochthonous origin, whereas lower values (< 1.3) indicate DOM of allochthonous origin (McKnight et al. 2001).

Given the oxic conditions of the study lakes, Fe^3+^ concentrations (measured in μg L^−1^ and reported as the negative log_10_ concentrations, pFe) were modeled using the chemical equilibrium modeling program Visual MINTEQ (version 4.0; https://vminteq.com). MINTEQ is a commercial version of MINEQL, a computer program developed in 1979 by Westall et al. (1979; 1994) to compute elemental speciation based on the thermodynamic chemical equilibria in aqueous systems. The speciation concentrations recorded are based on the total concentration of the element, the pH, redox state (pE), ionic strength, and temperature. The pH and temperature confounding parameters were measured using a YSI EXO-2 multiparameter sonde at a 1 m depth. Other potential confounding parameters were measured from water collected using a 1 m depth-integrated sampler to obtain a composite sample from the top meter of the water column. Water was then filtered through pre-combusted GF/F glass fiber filters. Potential confounding parameters included NO_3_^−^ and NH_4_^+^ (μg L^−1^). These samples were measured using Flow Injection Analysis (Lachat QuikChem 8500 FIA automated ion analyzer) following the US EPA Nitrate/Nitrite—Method 353.2 (Patton and Kryskalla 2011) and APHA Ammonia—Method 4500-NH3-B, H (Galvão et al. 2013). Sulphate (SO_4_^2−^) and chloride (Cl^−^) were measured with an Ion Chromatography (Dionex DX-600) using the US EPA Anion—Method 300.1 (Hautman and Munch 1997). Total dissolved Fe, calcium (Ca^2+^), and magnesium (Mg^2+^) were determined using an Agilent 7900 inductively coupled plasma mass spectrometry (ICP-MS), which allows for heightened sensitivity (i.e., lower detection limit) compared to colorimetric techniques (Mannio et al. 1995).

The concentration of siderophores (Sid) in the samples was measured using a SideroTec-HiSens Assay from Accuplex Diagnostics LTD, Ireland. This assay uses a colorimetric method to detect siderophores that bind to iron. In this method, Fe-binding siderophores react with a complex of chrome azurol S, Fe^3+^, and hexadecyltrimethylammonium bromide to produce a colored product (Schwyn and Neilands 1987). Standards (0–15 µM) were created and run in triplicate alongside water samples. Standards and samples (100 µL) were added to each well containing 100 µL of reagent and then gently mixed. The samples were incubated for 10 min at 37 °C and scanned on a microplate fluorometer using excitation wavelengths of 480 nm and emission at 525 nm. Water chemistry properties are provided for each lake in the Supplementary Information Table S3.

### Lake plankton analyses

Phytoplankton biomass was estimated using chlorophyll-*a* (Chl-*a*) and phycocyanin (PC) concentrations as proxies for total phytoplankton and cyanobacteria, respectively. Phytoplankton were concentrated using Whatman® GF/F filters and then frozen for subsequent analysis. The volume of water filtered ranged from 30 to 500 mL, depending on phytoplankton abundance. Chl-*a* was extracted using 90% acetone (v/v), while PC was extracted using a 0.1 M phosphate buffer (pH 6.8). The filters were suspended in 5 mL of solvent and mechanically disrupted using a bead beater (three cycles of 10 s) with 0.1 mm zirconium/silica beads. Chl-*a* extracts were stored at -20 °C and PC extracts at 4 °C for four hours before clarification by centrifugation (6,000 g for 5 min). The supernatant (1 mL) was filtered through a syringe 0.22 μm filter and measured using a spectrophotometer with a 1-cm path-length glass cuvette (Erratt et al. 2018). Chl-*a* concentrations were calculated using the method of Jeffrey and Humphrey (1975) at an absorbance wavelength of Ab 664 nm. PC concentrations were calculated using the method of Lawrenz et al. (2011) at an absorbance wavelength of Ab 620 nm. Chl-*a* and PC concentrations were reported in μg L^−1^.

Cyanobacteria taxonomic groups were analyzed using FlowCAM imagery at 20× magnification, which provides detailed taxonomic resolution down to the genus level (Jakobsen and Carstensen 2011). Surface water samples were immediately preserved with Lugol’s iodine solution and stored at 4 °C until analysis, which helped maintain cellular integrity. Samples were measured in triplicate, with 5000 images captured and subsequently sorted into major taxonomic groups, including *Aphanizomenon* spp., *Dolichospermum* spp., *Planktothrix* spp., and *Microcystis* spp., using the VisualSpreadsheet® Particle Analysis software and a freshwater algal taxonomic key (Bellinger and Sigee 2015). Relative abundance for major taxonomic groups was calculated by determining the percentage composition of each cyanobacteria genera within the total cyanobacteria count of each lake. Phytoplankton community composition was further validated using a BBE PhycoLab Analyzer, which performs a fluorometric assessment of Chl-*a* and major phytoplankton groups, including chlorophytes, cyanobacteria, diatoms, and cryptophytes. This method enables the evaluation of cyanobacterial dominance by quantifying their relative contribution to total Chl-*a* concentrations.

Hepatotoxins microcystin (MC) and nodularin, cyanobacterial toxins, were measured using an Enzo Life Sciences® MC (ADDA-specific) enzyme-linked immunosorbent assay (ELISA) kit. Lake water samples were filtered through Whatman® GF/F filters, followed by a freeze–thaw cycle (freezing at – 20 °C and thawing at room temperature). Thawed samples were immersed in 5 mL of 80% methanol (v/v) and mechanically disrupted using a bead beater (three cycles of 10 s) with 0.1 mm zirconium/silica beads to ensure cell lysis. Samples were centrifuged (10,000 g for 5 min), and the supernatant was analyzed. Before loading onto the microplate, a trial was conducted to determine the appropriate dilution factors (5–20 x) to ensure that MC concentrations fell within the range used to develop the standard curve. MC standards were read at Ab 450 nm on a Multiskan™ GO Microplate spectrophotometer (Thermo Scientific™) and used to construct a standard curve. The MC concentration of each sample was then determined using this curve (expressed in μg L^−1^).

Zooplankton grazing vs. filtering pressures were assessed, as these factors may influence phytoplankton biomass and diversity. Zooplankton community composition was visually evaluated using an Olympus CKX53 inverted microscope. Zooplankton samples were collected from the approximate center of each lake by repeatedly lowering and hauling a zooplankton net from the side of a boat or trolling the net behind the boat at low speeds. Sampling continued until sufficient material was collected for identification, with no predetermined sample volume. Collected zooplankton were stored in pre-rinsed wide-mouth HDPE bottles in a cool, dark environment to allow gut evacuation before being transferred to NASCO Whirl–Pak® bags for freezing within 12 h. Samples were thawed overnight in a refrigerator, gently mixed, and transferred to a Petri dish for identification. From each sample, 300 distinct individuals were identified without repetition using taxonomic sheets with representative images of zooplankton (Witty 2004). Deionized water was used for sample dilution when necessary. Cyclopoid copepods were classified as grazers, while calanoid copepods and Cladocera (*Daphnia* spp.) were classified as filter feeders, following the classifications of Bergström et al. (2024).

### Statistical analysis

Data were tested for normality using the Kolmogorov–Smirnov test to determine whether parametric or non-parametric statistical methods were appropriate. Because all data were non-normally distributed, natural log (ln) transformations were applied to Chl-*a* and PC, and a simple linear regression was performed between ln Chl-*a* and ln PC to assess the relative abundance of PC in the phytoplankton biomass. One-way analysis of variance on ranks (Kruskal–Wallis H test), followed by Dunn’s multiple pairwise comparison tests, were conducted to evaluate the significance of differences in: (a) Chl-*a*, PC, and MC under various limiting nutrient regimes; (b) mean Human Impact Index, agricultural cover (%), cropland cover (%), pastureland/grassland cover (%), and forest cover (%) under different limiting nutrient regimes; and (c) TP, TN, molar N:P ratio, Secchi depth, color, DOC, SUVA_254_, pFe, and siderophore concentration at different concentrations of PC. The above statistical analyses were performed using MATLAB R2019b. Spearman correlation matrices relating Chl-*a* and PC to physical and chemical properties were generated using R version 4.3.2.

## Results

### Total phytoplankton and cyanobacterial biomass

**Fig. 3:**
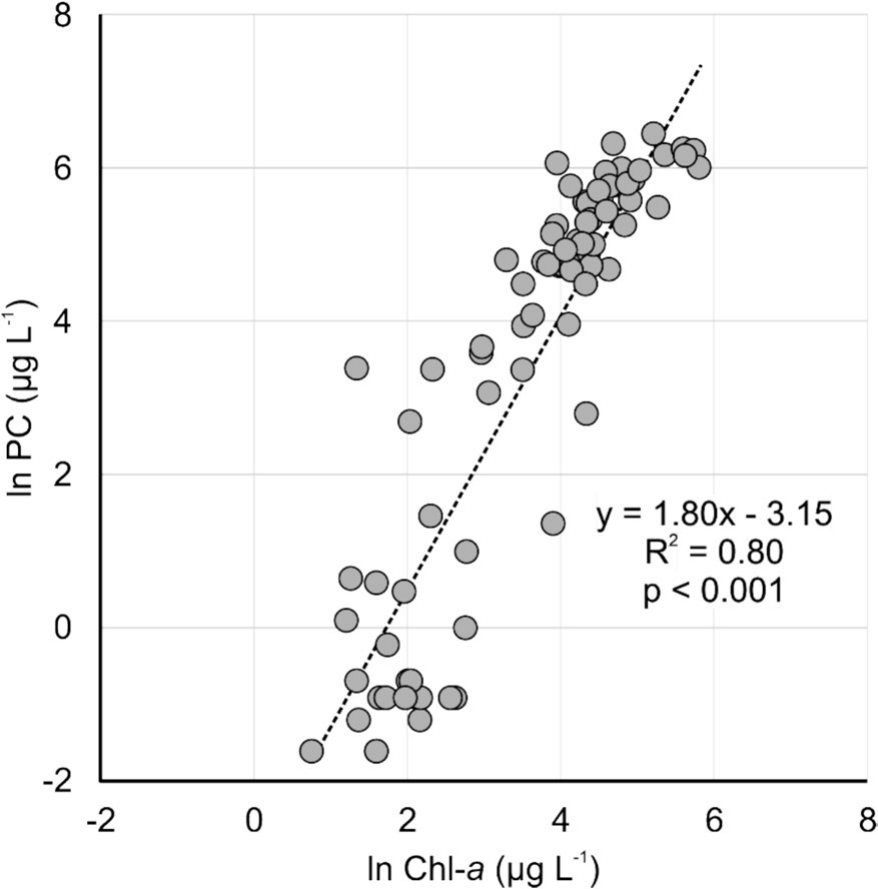
Relationship between proxies for total phytoplankton abundance (ln chlorophyll-*a* [Chl-*a*]) and cyanobacteria abundance (ln phycocyanin [PC])

The distribution of ln Chl-*a* across the lakes indicated a wide range of productivity, providing a robust basis for assessing factors influencing phytoplankton biomass (Fig. 3). A linear relationship was observed between ln Chl-*a* and ln PC across the lakes, showing a strong positive correlation (R^2^ = 0.80; p < 0.001) (Fig. 3). The strong positive correlation suggests that cyanobacteria are the dominant phytoplankton across this productivity spectrum. The BBE PhycoLab Analyzer results further support this finding, demonstrating a strong positive correlation between estimated phycocyanin (e.g., cyanobacteria via BBE) and extracted phycocyanin (r = 0.78, p < 0.0001) (Fig. S1).

### TP, TN vs. cyanobacterial biomass

**Fig. 4:**
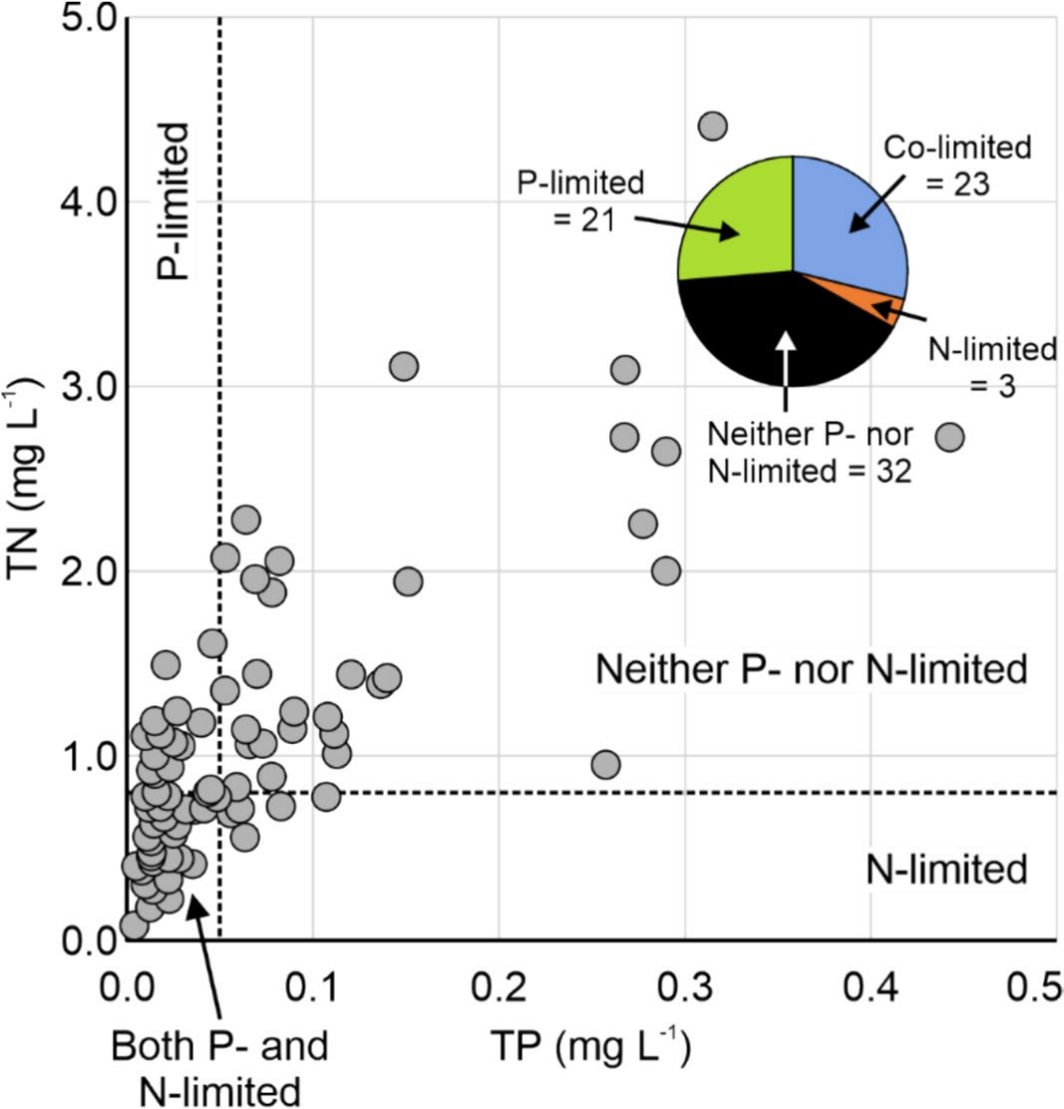
Lake total phosphorus (TP) vs. total nitrogen (TN). The vertical dotted line represents the threshold below which the lake is P-limited (i.e., TP < 0.05 mg L^−1^), and the horizontal dotted line represents the threshold below which the lake is N-limited (i.e., TN < 0.8 mg L^−1^) as proposed by Xu et al. (2015). The total number of lakes per nutrient limitation category is detailed in the pie chart

Analysis of macronutrient concentrations, specifically TP and TN, in relation to cyanobacteria biomass revealed that nutrient limitations in the lakes were more complex than the traditional P- or N-limited perspectives (Table S3). In Fig. 4, four quadrants are formed by TP and TN concentrations, representing the critical thresholds in cyanobacteria nutrient limitation. These quadrants identified 21 lakes (26.6%) as P-limited, 3 lakes (3.8%) as N-limited, 23 lakes (29.1%) as co-limited by both P and N, and 32 lakes (40.5%) as neither P- nor N-limited. Lakes classified as neither N- nor P-limited exhibited the highest Chl-*a* and PC concentrations (Table 1, p < 0.1).

**Table 1:** Means ± standard deviations of phytoplankton, cyanobacteria, and cyanobacterial toxin concentrations in lakes with different limiting nutrient regimes

Limiting nutrient	Chl-*a* (µg L^−1^)	PC (µg L^−1^)	MC (µg L^−1^)	Normalized MC MC (µg L^−1^)/PC (µg L^−1^) × 100
Co-limited	37.9 ± 44.06 A	71.4 ± 94.41 A	6.6 ± 12.65 AB	62.1 ± 80.46 n = 23^1^ 36.7 ± 82.06 n = 11^2^ 12.4 ± 15.99 n = 10^3^ A
P-limited	25.1 ± 28.99 A	40.2 ± 67.16 A	6.7 ± 19.85 A	44.9 ± 40.87 n = 21^1^ 16.2 ± 21.98 n = 7^2^ A
N-limited	54.2 ± 30.26 AB	155.2 ± 236.24 AB	65.3 ± 69.45 BC	111.0 ± 112.04 n = 3^1^ 46.8 ± 18.54 n = 2^3^ A
Neither P- nor N-limited	134.7 ± 88.84 B	279.0 ± 160.79 B	80.3 ± 97.69 C	51.7 ± 146.22 n = 32^1^ 51.7 ± 146.22 n = 32^2^ 26.2 ± 24.78^3^ A

^1^ Normalized MC concentration based on all data

^2^ Normalized MC concentration based on those lakes where MC was detected

^3^ One lake in this limiting nutrient regime had a remarkably high MC concentration, requiring further analysis; here, the normalized MC concentration is provided without this lake

Phytoplankton concentration is measured using chlorophyll-*a* (Chl-*a*) as a proxy, while cyanobacterial concentration is measured using phycocyanin (PC).

Cyanobacterial toxin concentrations are represented by microcystin (MC), which is presented as an absolute concentration and normalized levels.

Different letters indicate statistically significant differences (p < 0.1) between the limiting nutrient regimes.

### TP, TN vs. cyanobacteria community composition

The cyanobacterial community composition varied across the different nutrient regimes, with *Aphanizomenon* spp., *Dolichospermum* spp., *Planktothrix* spp., and *Microcystis* spp. dominating, albeit in various proportions within given lakes. Lakes with no nutrient limitations (neither P- nor N-limited) had lower abundances of *Aphanizomenon*, *Dolichospermum*, and *Microcystis* (but statistically significantly lower in *Aphanizomenon* and *Microcystis* only, p < 0.1). *Planktothrix* was less commonly found but most abundant in lakes without nutrient limitations. Zooplankton grazing pressure did not appear to influence phytoplankton biomass or community composition. The nutrient regimes exerted selective pressures on cyanobacterial community composition (Table 2).

**Table 2:** Means ± standard deviations of the composition of plankton across different nutrient limitation regimes

Limiting nutrient	No. lakes	A (%)	P (%)	D (%)	M (%)	Zooplankton grazers (%)	Zooplankton filterers (%)
Co-limited	23	70.6 ± 35.94 n = 11 AB	0 n = 0 A	48.9 ± 43.44 n = 11 A	54.1 ± 53.41 n = 4 A	49.5 ± 28.27 n = 23 A	46.5 ± 29.60 n = 23 A
P-limited	21	57.5 ± 39.59 n = 6 A	55.5 ± 42.78 n = 2 A	52.6 ± 44.04 n = 12 A	36.0 ± 55.50 n = 3 A	61.1 ± 24.49 n = 21 A	30.9 ± 25.69 n = 21 A
N-limited	3	64.3 ± 34.17 n = 3 AB	0n = 0 A	33.6 ± 34.32 n = 3 A	6.0 ± 0.00 n = 1 AB	28.4 ± 15.61 n = 3 A	27.3 ± 37.14 n = 3 A
Neither P- nor N- limited	32	57.0 ± 36.18 n = 27 B	79.5 ± 42.69 n = 5 A	25.0 ± 31.86 n = 18 A	32.1 ± 34.33 n = 23 B	41.8 ± 29.80 n = 32 A	42.4 ± 29.53 n = 32 A

The breakdown of phytoplankton composition does not sum to 100% because not all study lakes are included in the genera analysis.

The percentages reflect the relative abundance within the subset of lakes where each genus was observed, not the total number of study lakes in each nutrient regime.

For example, *Aphanizomenon* was present in 11 of the 23 Co-limited lakes, and the reported percentage (70.6 ± 35.94%) represents its abundance in those lakes.

For phytoplankton, the means ± standard deviations of each taxonomic group are presented in lakes in which they are found, along with the number of lakes where they are found (A = *Aphanizomenon* spp.; P = *Planktothrix* spp.; D = *Dolichospernum* spp.; M = *Microcystis* spp.)

Since all lakes contain both grazers and filterers, only the means ± standard deviations of these functional groups are provided for zooplankton.

Different letters indicate statistically significant differences (p < 0.1) between the limiting nutrient regimes.

### TP, TN vs. cyanobacteria toxins

MC was detected across all nutrient regimes. The lowest MC concentrations were observed in co-limited and P-limited lakes. In contrast, lakes with no nutrient limitation exhibited the highest MC concentrations, approximately 12-fold greater than in co-limited and P-limited lakes. N-limited lakes had the highest normalized MC concentrations when normalized to cyanobacterial biomass. Co-limited and P-limited lakes had the lowest normalized MC concentrations. Lakes without nutrient limitations had moderate normalized MC concentrations. Three lakes were identified as outliers with extremely high normalized MC concentrations and were excluded from further analysis (Table 1). Compared to the range in normalized MC concentrations when these three lakes were excluded (0.31–188%), Beaver Mines had a normalized MC concentration of 280% (PC = 3.90 μg L^−1^, MC = 11.0 μg L^−1^), Elinor Lake had a normalized MC concentration of 240% (PC = 16.30 μg L^−1^, MC = 39.04 μg L^−1^), and Shell Lake had the highest normalized MC of 842%, despite a small concentration of PC (PC = 1.00 μg L^−1^, MC = 8.42 μg L^−1^).

### Human impact influences the nutrient limitation regime

Human activities within the contributing catchment area of the lakes were correlated with the nutrient regimes of the lakes. The Human Impact Index is an integer index, with a value of 0 indicating low levels of human modification (i.e., forests), a value of 1 where human modification of land cover is moderate (i.e., pastureland or grassland, forest loss), or a value of 2 where human modification of land cover is high (i.e., cropland, urban development, mines, oil activities). Lakes with no nutrient limitations exhibited higher levels of human impact, as indicated by a higher mean Human Impact Index (Table 3). These lakes also had a higher percentage of agricultural land cover and a lower percentage of forest cover. The significant differences in human impact across the nutrient regimes suggest that human activities in the contributing catchment areas increase the availability of P and N in the lakes, thereby increasing cyanobacterial biomass and changing nutrient limitation status (Table 3).

**Table 3:** Means ± standard deviations of human impact in contributing catchment areas as characterized by the mean Human Impact Index (ranging from 0 to 2) and the percent agricultural cover (cropland plus pastureland), cropland, pastureland and grassland, and forest cover across different limiting nutrient regimes

Limiting nutrient	Human impact index [0–2]	Agricultural cover (%)	Cropland cover (%)	Pastureland/Grassland cover (%)	Forest cover (%)
Co-limited	0.6 ± 0.58 A	18.1 ± 28.01 A	12.5 ± 23.28 A	5.7 ± 10.25 A	44.4 ± 28.65 A
P-limited	0.7 ± 0.51 A	18.5 ± 22.05 A	9.7 ± 16.32 A	8.8 ± 9.63 AB	38.8 ± 25.81 A
N-limited	0.2 ± 0.05 AB	5.2 ± 4.92 AB	0.3 ± 0.37 AB	4.9 ± 4.61 AB	62.7 ± 8.20 A
Neither P- nor N-limited	1.1 ± 0.55 B	40.9 ± 25.90 B	27.5 ± 25.18 B	13.4 ± 10.09 B	27.0 ± 26.26 A

Different letters indicate statistically significant differences (p < 0.1) between the limiting nutrient regimes.

### Physical factors influencing cyanobacterial biomass under different nutrient limitation regimes

**Fig. 5:**
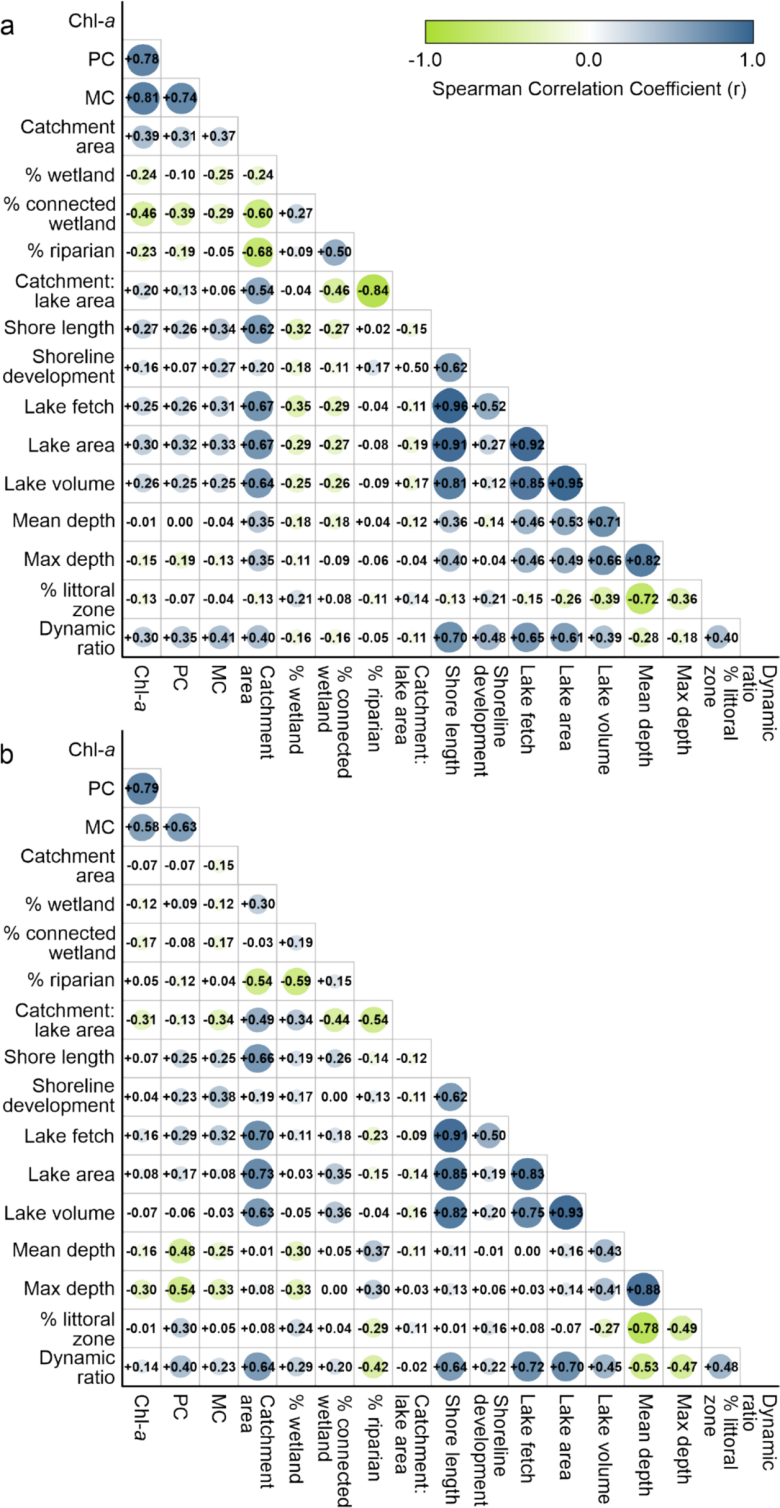
Spearman correlation coefficient matrices showing relations between chlorophyll-*a* (Chl-*a*), phycocyanin (PC), and microcystin (MC) concentrations and lake physical properties in (a) nutrient-limited lakes (phosphorus (P)-, nitrogen (N)-, and P and N co-limited) and (b) neither P- nor N-limited lakes. Catchment area = catchment land area; % wetland = percent wetland area in catchment; % connected wetland = percent wetland area connected via drainage networks to lake in catchment; % riparian = percent riparian area in catchment; shore length = shoreline length; % littoral zone = percent littoral zone in lake

The physical factors were not strongly associated with Chl-*a*, PC or MC (Fig. 5).

In lakes limited by P, N, or co-limited by P and N (Fig. 5a), larger catchment areas were positively correlated with Chl-*a* (r =  + 0.39), indicating catchment sources of nutrients for phytoplankton, whereas wetland connectivity to surface waters was negatively correlated to Chl-*a* (r =  − 0.46) and PC (− 0.39), indicating catchment sinks of nutrients for phytoplankton. Dynamic ratio was positively correlated with Chl-*a* (r =  + 0.30), PC (r =  + 0.31), and MC (r =  + 0.41), indicating within lake sources of nutrients for phytoplankton.

In lakes that were neither P- nor N-limited (Fig. 5b), lake mean (r = -0.48) and maximum (r = − 0.54) depths were negatively correlated with PC, whereas lake dynamic ratio (r =  + 0.40) was positively correlated with PC (Fig. 5b). These correlations suggest that PC is elevated in shallower lakes, where there is a higher probability of resuspension of nutrient-rich sediments from the lake bottom.

### Chemistry factors influencing cyanobacterial biomass under different nutrient limitation regimes

**Fig. 6:**
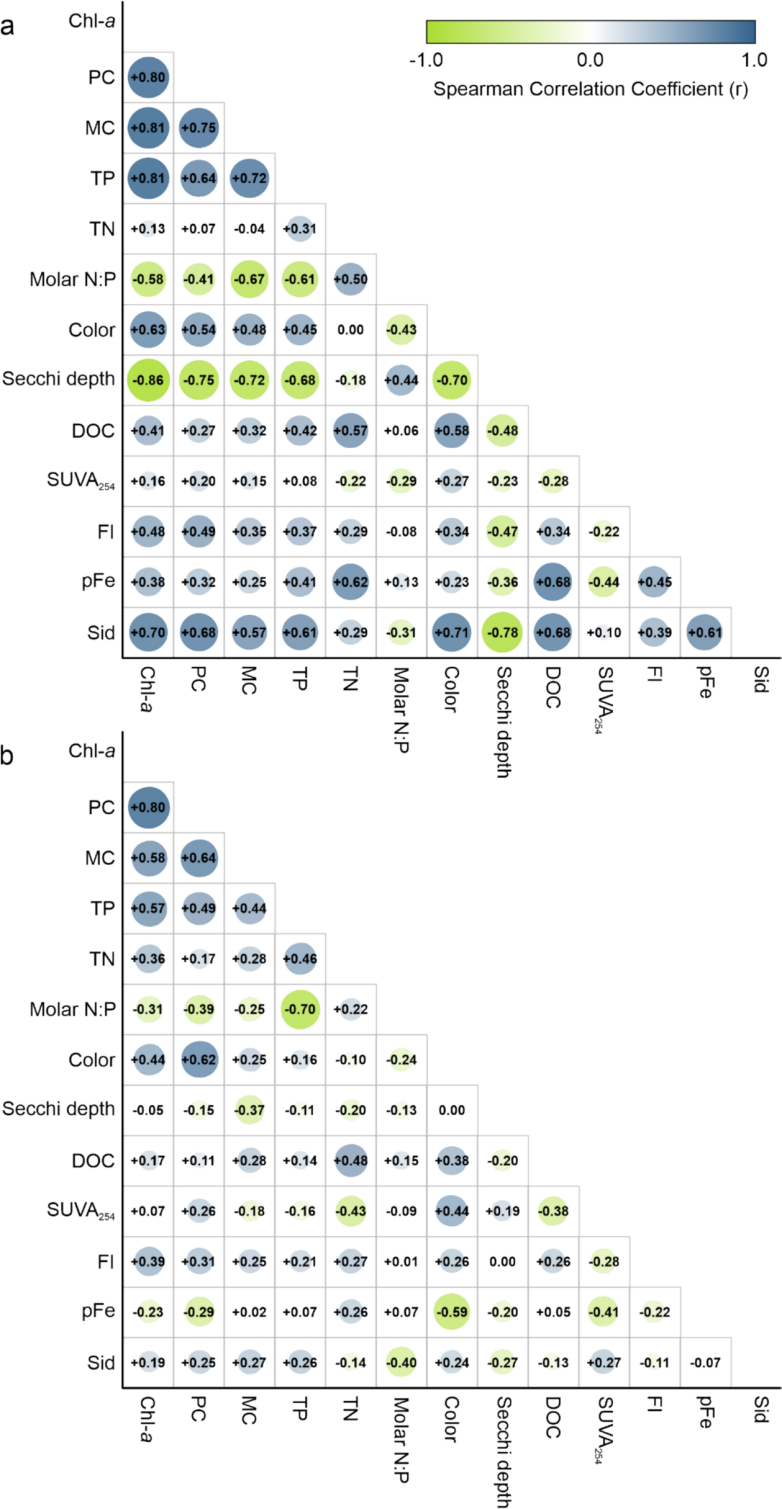
Spearman correlation coefficient matrices showing relations between chlorophyll-*a* (Chl-*a*), phycocyanin (PC), and microcystin (MC) concentrations and water chemistry properties in **a** nutrient-limited lakes (phosphorus (P)-, nitrogen (N)-, and P and N co-limited) and **b** neither P- nor N-limited lakes. TP  = total P; TN  ﻿= total N, DOC ﻿= dissolved organic carbon concentration, SUVA_254_ ﻿= specific ultraviolet absorbance at 254 nm, FI ﻿= fluorescence index, pFe  ﻿= -log_10_[ferric Fe], Sid ﻿= siderophore concentration

Lakes with neither P nor N nutrient limitations displayed a wide range of PC concentrations. These lakes were further categorized into six sub-groups based on their PC concentrations, and various environmental factors influencing cyanobacterial community outcomes were analyzed (Table 4, Fig. 6). The main observations are summarized below:

**Table 4:** Means ± standard deviations of phycocyanin (PC), total phosphorus (TP), and total nitrogen (TN) concentrations, molar N:P ratio, color, Secchi depth, dissolved organic carbon (DOC) concentration, specific ultraviolet absorbance at 254 nm (SUVA_254_), modeled pFe where pFe is the modeled available ferric Fe (pFe = -log_10_[Fe^3+^]), and siderophore (Sid) concentration in lakes classified as neither P- nor N-limited. Means ± standard deviations were calculated for each classified PC concentration sub-group

PC sub-group (µg L^**−1**^**)**	PC (µg L^**−1**^**)**	TP (µg L^**−1**^**)**	TN (µg L^**−1**^**)**	Molar N:P	Color (TCU)	Secchi depth (m)	DOC (mg L^**−1**^**)**	SUVA_254_ (L mg C^−1^ m^−1^)	FI (unitless)	pFe (unitless)	Sid (nmol mL^**−1**^**)**
< 100	27.4 ± 25.63 A	89.8 ± 40.5 A	1675.0 ± 466.7 A	46.5 ± 22.6 A	26.4 ± 2.6 A	1.5 ± 1.2 A	22.6 ± 11.1 A	1.5 ± 0.2 A	1.8 ± 0.1 A	23.6 ± 2.7 A	250.2 ± 56.4 A
100–200	136.5 ± 30.17 A	113.4 ± 69.4 A	2093.9 ± 988.2 A	45.82 ± 21.5 A	31.5 ± 23.3 A	0.7 ± 0.4 A	23.7 ± 10.7 A	1.6 ± 0.6 A	1.7 ± 0.1 A	24.2 ± 2.1 A	578.4 ± 179.1 A
200–300	255.5 ± 28.59 AB	106.0 ± 83.6 A	1476.4 ± 604.8 A	41.6 ± 29.3 A	36.6 ± 25.4 A	0.8 ± 0.4 A	21.9 ± 6.4 A	1.7 ± 0.6 A	1.7 ± 0.1 A	23.8 ± 2.1 A	568.0 ± 94.0 A
300–400	344.2 ± 32.76 B	118.9 ± 65.5 A	1364.4 ± 455.6 A	30.7 ± 16.3 A	54.8 ± 44.0 A	0.7 ± 0.2 A	17.9 ± 5.0 A	1.9 ± 0.7 A	1.8 ± 0.1 A	22.8 ± 2.2 A	604.5 ± 92.8 A
400–500	451.7 ± 41.13 B	292.7 ± 162.2 A	3200.0 ± 1667.6 A	26.5 ± 10.1 A	58.2 ± 34.6 A	0.7 ± 0.3 A	34.2 ± 13.5 A	1.3 ± 0.3 A	2.0 ± 0.1 A	23.4 ± 0.4 A	568.5 ± 160.8 A
500 +	550.3 ± 55.79 B	246.6 ± 64.2 A	2678.8 ± 407.1 A	24.9 ± 5.0 A	72.4 ± 57.5 A	0.8 ± 0.3 A	27.3 ± 7.7 A	1.9 ± 0.5 A	1.8 ± 0.0 A	24.0 ± 1.1 A	592.6 ± 76.4 A

Different letters indicate significant differences (*p* < 0.1) between classified PC concentration sub-groups

*TP, TN, and N:P Correlations:* For co-, P-, or N-limited lakes, the correlation coefficient between TP and TN was + 0.31, and between TP and molar N:P was − 0.61 (Fig. 6a). For neither P- nor N-limited lakes, the correlation coefficient between TP and TN was higher at + 0.46, while between TP and molar N:P, it was higher at − 0.71 (Fig. 6b). TP and TN concentrations generally increased, but the molar N:P ratio decreased as cyanobacterial biomass increased from a mean N:P = 46.5 in sub-group < 100 (i.e., lowest concentrations of PC) to a mean N:P = 24.9 in sub-group 500 + , indicating a trend towards N-stress (Table 4).

*Catchment “fingerprint” on the lakes*: The correlation coefficients between chemical factors associated with catchment support of cyanobacterial populations (i.e., color, DOC, SUVA_254_, and FI) and PC did not indicate a clear origin pattern among the lakes of different nutrient regimes (Fig. 6). However, in the neither P- nor N-limited lakes, color, DOC, and SUVA_254_ generally increased as PC concentrations rose, reflecting a shift towards higher aromatic content in DOC. FI showed no trend, with values ranging from a mean of 1.8 to 2.0, indicating a combination of allochthonous and autochthonous DOC sources in all lakes (Table 4).

*Modeled Fe correlations*: No correlation was found between modeled Fe^3+^ (pFe) (pFe = − log_10_[Fe^3+^]) and PC concentrations in the study lakes (Fig. 6). All lakes had pFe values between pFe18 and pFe26, with concentrations of most lakes above pFe20. At such low levels, there is insufficient Fe to meet the needs of cyanobacterial cells. In co-, P-, and N-limited lakes, the correlation coefficient between pFe and PC was − 0.32 (Fig. 6a). In neither P- nor N-limited lakes, the correlation coefficient between pFe and PC was higher at − 0.39 (Fig. 6b). In response to such low pFe levels, cyanobacteria may produce siderophores to bind and transport pFe, maintaining a biologically available pool of Fe within the epilimnetic cyanobacterial community.

**Fig. 7:**
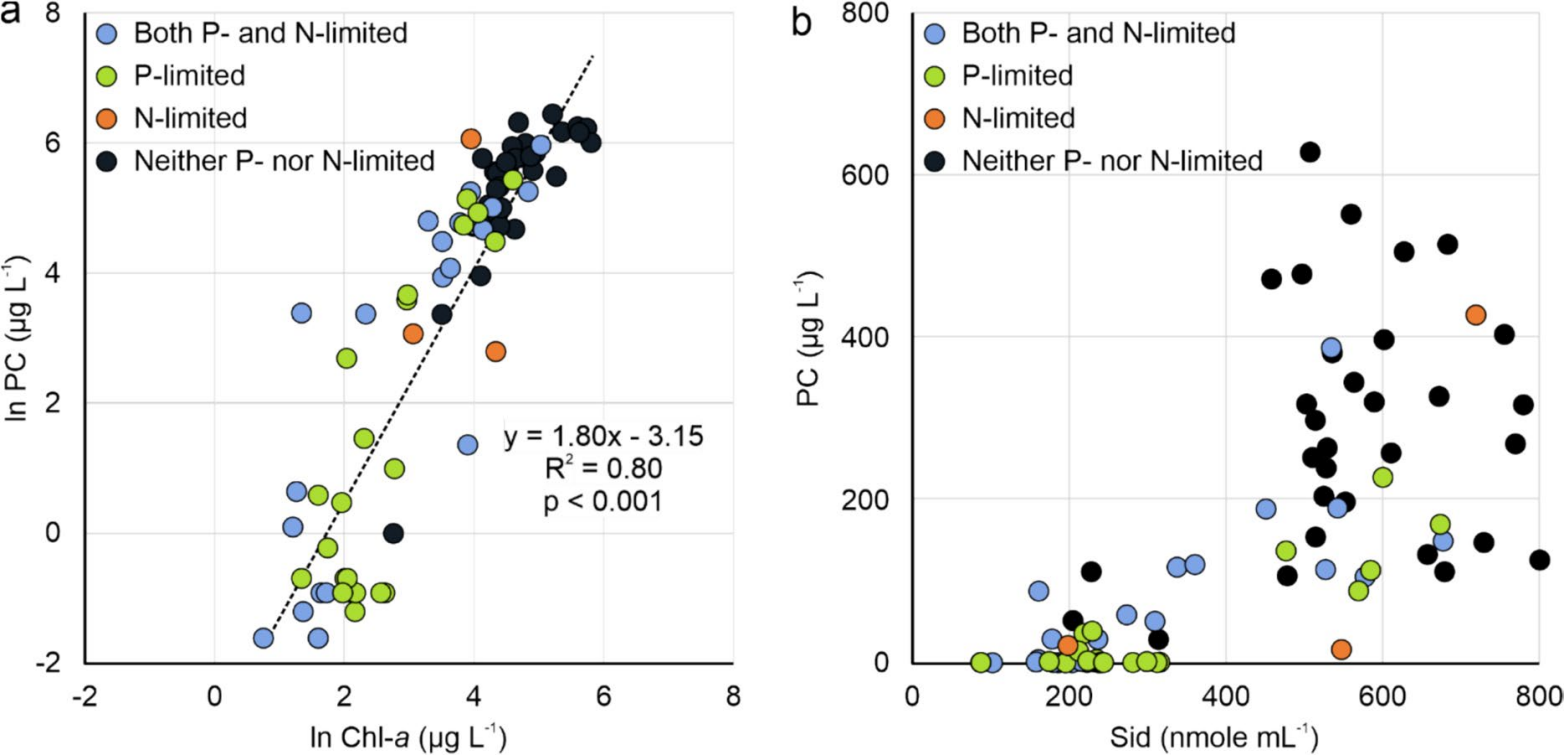
**a** The relationship between ln phycocyanin (PC) and ln chlorophyll-*a* (Chl-*a*) with nutrient limitation regimes identified. **b** A plot of cyanobacteria biomass (PC) vs. siderophore (Sid) concentration. The symbols correspond to lakes under different nutrient limitations: co-limited, phosphorus (P)-limited, nitrogen (N)-limited, and neither P- nor N-limited

*Siderophore production*: All lakes recorded the presence of siderophores, with concentrations ranging from 100 to 800 nmol mL^⁻1^. Co-limited and P-limited lakes tended to have lower siderophore concentrations than lakes that were neither P- nor N-limited. However, the limited number of N-limited lakes prevented the establishment of a definitive pattern. PC concentrations were minor when siderophore concentrations were below 400 nmol mL^⁻1^ and largest when siderophore concentrations exceeded 400 nmol mL^⁻1^ (Fig. 7).

Siderophore production is unnecessary in lakes where Fe is not the limiting nutrient. For example, in co- and P-limited lakes, siderophore concentrations were low—co-limited lakes had mean ± standard deviation siderophore concentrations of 305.1 ± 163.63 nmol mL^−1^, and P-limited lakes had siderophore concentrations of 309.1 ± 18.08 nmol mL^−1^. Nutrient-limited lakes (P-, N-, and P and N co-limited) had a lower mean ± standard deviation siderophore concentration (318.47 ± 173.5 nmol mL^−1^) compared to lakes that were neither P- nor N-limited (551.91 ± 151.58 nmol mL^−1^). The correlation coefficient between siderophore and PC concentrations in nutrient-limited lakes was + 0.68 (Fig. 6a). Correlation coefficients between siderophore concentration and factors linked to catchment support of cyanobacterial populations were color (r =  + 0.71) and DOC concentrations (r =  + 0.68) (Fig. 6a) were high in nutrient-limited lakes. This suggests that Fe-binding siderophores, which also show correlations with macronutrients such as TP (r =  + 0.61) and TN (r =  + 0.29), may originate from catchment runoff (Fig. 6).

In lakes that were neither P- nor N-limited, cyanobacteria may be prolific siderophore producers, with siderophore concentrations averaging 551.9 ± 151.58 nmol mL^−1^. While it is challenging to determine whether cyanobacteria are the sole contributors to siderophore production, given the potential for other microbes to produce siderophores and the high microbial diversity associated with cyanobacteria blooms, there is strong evidence supporting that, in Fe-limited environments, select cyanobacterial genera can synthesize these Fe-ligands to mitigate stress (Wilhelm and Trick 1994).

While no significant trend in siderophore production was observed in these lakes, lakes with the lowest PC concentrations (sub-group < 100) had siderophore concentrations averaging 250 nmol mL^−1^. In contrast, lakes with higher PC concentrations (sub-groups 100–500 +) exhibited average siderophore concentrations ranging from 450 to 600 nmol mL^−1^, though the range for discrete samples was much broader (Table 4). The correlation coefficient between siderophore concentration and PC in these lakes was lower at + 0.25 (Fig. 6b). Correlations between siderophore concentration and factors associated with catchment support of cyanobacterial populations were low. Siderophores may be produced to maintain low but constant pFe concentrations in lake communities and facilitate the limited transport of N into cyanobacterial cells, as reflected by the molar N:P ratio (Table 4). However,siderophores may be produced in excess of Fe^3+^ in the environment or the cell’s needs (Fig. 7).

## Discussion

This study examined the complex relationships between lake chemistry and cyanobacterial biomass in the Prairie Pothole Region of western Canada. Our focus was on understanding how the availability of critical nutrients—P, N, and Fe—influences cyanobacterial biomass. Our findings consistently showed that cyanobacteria were dominant in all sampled lakes despite significant variability in the lakes’ physical and chemical properties. The cyanobacterial communities primarily consisted of potentially toxin-producing *Aphanizomenon* spp., *Dolichospermum* spp., *Planktothrix* spp., and *Microcystis* spp. We tested three hypotheses to identify the environmental factors that drive cyanobacterial biomass in these lakes, which is necessary to predict and effectively mitigate cyanobacterial dominance.

### H1

Cyanobacteria in all lakes are P-limited

Traditionally, P was considered the critical limiting nutrient in many freshwater ecosystems, especially in temperate regions that were not undergoing cultural eutrophication (Schindler 1977; Downing et al. 2001). The limitation of N in freshwaters has gained increased attention recently (Paerl et al. 2016). In environments deficient in macronutrients, adding the missing element, either P or N, typically leads to a rapid increase in algal growth due to these nutrient constraints. In contrast, the response of primary producers to the addition of macronutrients may be less effective if the availability of essential micronutrients, such as Fe, restricts the cells’ ability to assimilate macronutrients efficiently (Wetzel 2001).

Here, we found that only 59.5% of the study lakes were controlled by macronutrients, with most of these being P-limited (26.6%) or co-limited by P and N (29.1%), with the remaining being N-limited (3.8%) (Table 2). These macronutrient-limited lakes were situated in catchments that included large percentages of forest cover and small percentages of human-modified lands (croplands and pasturelands) (Table 3). When catchment processes are considered, these macronutrient-limited lakes likely have relatively small nutrient loading from the catchment to the lake, contributing to P and/or N limitation.

Factors influencing external nutrient loading processes were correlated to phytoplankton biomass in macronutrient-limited environments. Larger catchments with smaller wetlands connected via surface drainage networks to the lakes likely had larger nutrient loads that promoted phytoplankton growth (Erratt et al. 2023; Whitfield et al. 2024). Further, internal nutrient loading processes likely provided more significant nutrient concentrations that promoted cyanobacterial dominance and microcystin concentrations. A higher dynamic ratio increases the likelihood of nutrient resuspension from bottom sediments, supporting internal loading processes (Søndergaard et al. 2001). These shallow lakes also warm quickly, favoring potentially toxigenic cyanobacteria, which have higher temperature preferences compared to eukaryotic algae (O’Neil et al. 2012). Therefore, H1—that cyanobacteria in the lakes are macronutrient-limited—is only partially supported, as macronutrients controlled 59.5% of study lakes, but 40.5% were not.

While the universal application of macronutrient thresholds from Xu et al. (2015) requires caution due to variability in lake nutrient regimes, these thresholds offer valuable insights into nutrient-enriched environments. MacKeigan et al. (2023) report that 82% of lakes in the PPR have a trophic status consistent with the eutrophic state of Lake Taihu (Xu et al. 2015). Despite its larger size (2250 km^2^), Lake Taihu shares key characteristics with many study lakes, particularly its shallow nature and susceptibility to agricultural runoff, leading to comparable nutrient regimes. Taihu’s mean depth is 1.9 m (Xu et al. 2015; Paerl et al. 2020), while lakes in our study have a greater mean depth of 5.1 m (Erratt et al. 2023; MacKeigan et al. 2023). Cyanobacterial dominance is widespread across the Canadian Prairie provinces, with phytoplankton biomass composed of approximately 62% cyanobacteria (MacKeigan et al. 2023), closely mirroring the cyanobacterial dominance in Lake Taihu (Xu et al. 2015). Our study area is recognized as a hotspot for cyanobacterial biomass (MacKeigan et al. 2023). The elevated trophic status, shallow bathymetry, and high cyanobacterial biomass suggest that the study lakes functionally resemble Lake Taihu.

### H2

Cyanobacteria in lakes situated in agriculturally dominated landscapes are not limited by P or N.

Here, we predicted that cyanobacteria in lakes with substantial human impact within the contributing catchments that drain into the lakes would be classified as neither P- nor N-limited lakes. Of the study lakes, 40.5% did not conform to the traditional nutrient limitation model supported by H1.

These lakes, neither P- nor N-limited, are characterized by the largest cyanobacterial biomass with a mean ± standard deviation Chl-*a* concentration of 134.7 ± 88.84 µg L^−1^ and PC concentration of 279.0 ± 160.79 µg L^−1^ (Table 1). In contrast to the macronutrient-limited lakes, neither P- nor N-limited lakes had catchments with a high mean Human Impact Index value (Table 3), which indicates a higher proportion of the catchment area dominated by human-modified lands. Most P and N contributions in the PPR typically come from agricultural sources. This is due to the region’s extensive agrarian land cover, where runoff from fertilizers and manure significantly contributes to nutrient loading in lakes and subsequently fuels cyanobacterial growth (Erratt et al. 2023; MacKeigan et al. 2023). Atmospheric deposition contributes to nutrient levels, but its impact is generally less than the direct contributions from agricultural activities (Baulch et al. 2019). One measure of catchment nutrient supply was the presence of terrestrial dissolved organic matter (DOM). The highest concentrations of Chl-*a* and PC corresponded with increased color, increased DOC concentration, higher SUVA_254_, and slightly higher FI establishing the catchment-lake nexus (Creed et al. 2018; Senar et al. 2021) (Table 4).

Terrestrial-derived DOM functions as a nutrient vector, introducing growth-constraining nutrients into aquatic environments (Creed et al. 2018). Nutrients applied to agricultural catchments may bind to these DOM complexes and be transported from land to lake during periods of heightened hydrological connectivity. Mean SUVA_254_ values below 3 L mg C^−1^ m^−1^ indicate that the DOM supplied to the lakes is biologically available to the phytoplankton community, making nutrients more readily accessible to fuel cyanobacterial growth (Weishaar et al. 2003). These labile DOM contributions form nutrient-DOM complexes enriched with additional P and N supplemented to agricultural catchments. The nutrients bound to these DOM complexes are readily assimilated, contributing to elevated cyanobacterial biomass (Wilson and Xenopoulos 2009). In lakes that were neither P- nor N-limited, there was a negative correlation exists between SUVA_254_ and pFe (− 0.41, Fig. 5); Fe imparts a yellow or orange coloration to water (Poulin et al. 2014), but the overall low Fe content in our study lakes likely results in a negligible contribution of Fe to SUVA_254_ measurements. Further physical modifications within these lakes, such as enhanced color, can create conditions that favor cyanobacteria over their eukaryotic competitors. For example, increased color can restrict light penetration, with lower light levels favoring cyanobacteria that possess accessory pigments suited for low-light conditions (e.g., PC) (Stomp et al. 2007; Erratt et al. 2021). Therefore, H2 is supported based on the high cyanobacterial biomass and the active supply of macronutrients from the human-modified catchment.

### H3

Cyanobacteria in lakes that are neither P- nor N-limited cannot benefit from added P or N due to the lack of the micronutrient Fe.

Here, we predicted that cyanobacteria communities inhabiting lakes with total TP and TN levels above the critical thresholds (i.e., 0.05 mg L⁻^1^ TP and 0.8 mg L⁻^1^ TN, as suggested by Xu et al. 2015) receive a constant flux of P and N at a rate where the supply of Fe limits growth and total macronutrient consumption. Additionally, we predicted that cyanobacteria would respond to low Fe conditions by producing and using siderophores to scavenge Fe.

The modeled pFe was generally constant across the study lakes, with low pFe values (> pFe 18) indicating Fe stress, which limits NO_3_^−^ assimilation and N_2_-fixation (Kerry et al. 1988; Wilhelm and Trick 1994). In response to these conditions, cyanobacteria produce siderophores to scavenge Fe, facilitating nutrient uptake and cell replication (Årstøl and Hohmann-Marriott 2019). Siderophores have a high affinity for Fe^3+^ and form soluble Fe^3+^ complexes, facilitating active transport and subsequent reduction to Fe^2+^ into the cyanobacterial cell (Wilhelm and Trick 1994; Wilhelm et al. 1998; Molot et al. 2014). The cells also induce a specific Fe-siderophore transport system to effectively transport Fe into the cells. Cyanobacteria studied across various lake environments ranging from oligotrophic to hypereutrophic have all shown the ability to produce siderophores to access Fe in limiting environments (Sorichetti et al. 2016).

**Fig. 8:**
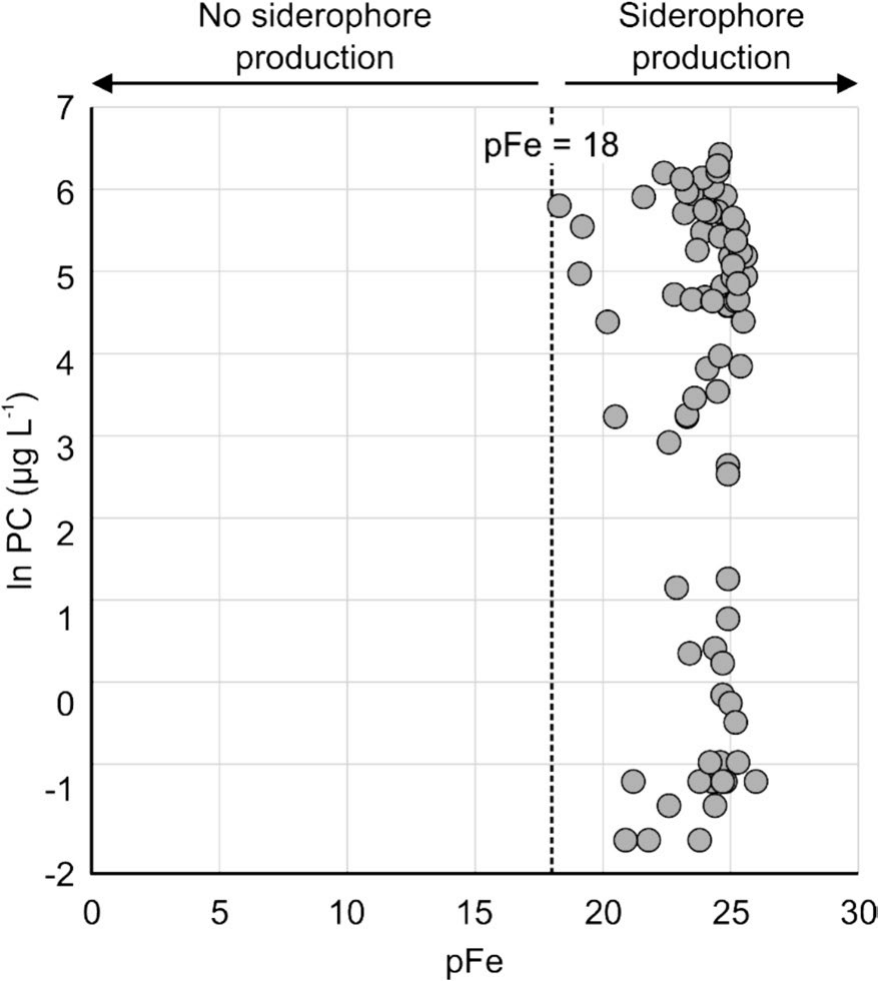
Plot of ln phycocyanin (PC) vs. pFe, where pFe is the modeled available ferric Fe (pFe = − log_10_[Fe^3+^]). pFe = 18 is the threshold above which there is no siderophore production and below which there is siderophore production (Kerry et al. 1988)

Of the study lakes, 40.5% were neither P- nor N-limited, with cyanobacterial growth insufficient to deplete both macronutrients. We propose that Fe was the limiting factor in these lakes. Our modeled levels of pFe indicate that these lakes have Fe at levels that are significantly growth-limiting (i.e., > pFe 18) (Fig. 8). Fe has two dominant supply routes into cyanobacteria biomass: supply from the catchment (Xiao et al. 2013) and supply from the sediments (Molot et al. 2014). There is no correlation with DOM acting to supply Fe (correlation coefficient =  + 0.05, Fig. 5b), indicating that sediment Fe might be the supply (Molot et al. 2014; Orihel et al. 2015). We observed that neither P- nor N-limited lakes exhibited strong correlations with bathymetric features, indicating that shallower systems favor cyanobacterial growth (e.g., PC vs. dynamic ratio =  + 0.40). Lakes with a higher dynamic ratio are more susceptible to wind-driven mixing, which can significantly impact nutrient dynamics. This increased surface area facilitates more frequent wind-induced mixing, particularly in shallow lakes, and plays a crucial role in resuspending nutrients from bottom sediments. In these well-mixed environments, the redistribution of resuspended sediments provides a continuous supply of nutrients, supporting cyanobacterial blooms even in the absence of significant external inputs (Orihel et al. 2015).

Moreover, Fe deficiencies are likely to persist in these environments due to the lack of anoxic conditions, which promotes Fe-retention to P and prevents the release of Fe from sediments (Molot et al. 2014). Prairie lakes, which are typically shallow and often exhibit a polymictic mixing regime, may experience more pronounced thermal stratification due to rising temperatures and reduced wind activity associated with climate change (Orihel et al. 2015; Woolway et al. 2019). This shift toward a more stabilized stratification regime is expected to amplify nutrient pulses, such as Fe, from sediments in these shallow water bodies due to heightened likelihood of deoxygenation events in the hypolimnion (Søndergaard et al. 2001; Welch and Cooke 2005). The strengthening of thermal stratification in the temperate lakes (Jane et al. 2021; Woolway et al. 2021) may promote Fe loading from sediments, enabling cyanobacteria to utilize surplus macronutrients and potentially exacerbate concerns related to cyanobacterial blooms (Molot et al. 2014).

Lakes with a “neither P- nor N-limited” regime produced and released significant levels of siderophores, indicating that the community was Fe-limited and could only process new P and N at the rate of Fe supply (Table 4, Fig. 7). (Table 4, Fig. 7). Unlike macronutrients with specific and regulated transport systems, siderophore production was not dependent on cell biomass. These lakes would be considered “slow metabolism” systems, as cyanobacterial growth is stalled due to the lack of Fe. Without metabolic feedback from the acquisition of Fe, cells do not down-regulate siderophore production. The environment external to the cells remains rich with transporters (high potential), with too little Fe to transport (low performance). The result of siderophore-mediated P and N transport is that the cells lack robust acquisition of the macronutrients, keeping P and N drawdown to a minimum. As a result, a long-standing stock of cyanobacteria persists, stalled by Fe limitation. Neither P- nor N-limited lakes exhibited the highest MC content (Table 1). Among the various theories proposed for the function of MC, one gaining support is its role as a Fe acquisition molecule, facilitating growth under Fe-starved conditions (Omidi et al. 2018). In nutrient-replete environments (i.e., neither P- nor N-limited lakes), cyanobacteria may face less competition for P and N but might still experience competition for other micronutrients. High MC production in these lakes could suggest that cyanobacteria prioritize toxin production as an allelopathic response to access limited resources (i.e., Fe). Alternatively, MC may function as a Fe-chelating molecule to aid Fe acquisition (Omidi et al. 2018).

Compared to P-, N-, and co-limited systems, the weaker correlation between siderophore and MC concentrations (+ 0.27) in neither P- nor N-limited lakes may be explained by MC functioning more as an allelopathic mechanism. In nutrient-replete environments with high macronutrient availability and biomass, cyanobacteria face multiple factors influencing metabolism (e.g., light, trace metals), potentially leading to a decoupling between siderophore and MC production. In neither P- nor N-limited lakes, the pressure to acquire macronutrients is reduced, allowing cyanobacteria to shift their metabolic focus. Rather than prioritizing nutrient acquisition, they may invest in allelopathic mechanisms to outcompete other organisms for more limited resources, such as trace metals like Fe. In contrast, the moderate correlation between siderophore and MC concentrations (+ 0.57) in P-, N-, and co-limited systems could be attributed to a stress response driven by nutrient scarcity. Nutrient sequestration mechanisms (such as MC’s potential role in Fe acquisition) (Ceballos-Laita et al. 2017) would likely take precedence over allelopathy, as the primary survival strategy would be to secure the scarce macronutrients essential for growth. Therefore, H3 is supported based on the high levels of the inducible siderophores in the lake communities.

Lake management strategies typically focus on controlling macronutrient (P and N) inputs to mitigate eutrophication and cyanobacterial blooms, as these external inputs are generally more easily regulated (Schindler and Vallentyne 2008; Paerl et al. 2016; Erratt et al. 2022). In systems where neither N nor P is limiting (and Fe limitations persist), reductions in macronutrient inputs may not result in immediate declines in biomass, highlighting the need to refine expectations regarding the effectiveness and timeline of management efforts. This study underscores the crucial role of micronutrients, particularly Fe, in regulating cyanobacterial dynamics in freshwater environments, emphasizing the potential of Fe as a critical factor in cyanobacteria management (Molot et al. 2014). A key challenge in regulating Fe lies in its availability within lakes, which is primarily governed by natural hydrological and biogeochemical processes, complicating direct management actions. Facilitating the identification of Fe-limited systems would ensure that lake management strategies are tailored to the specific biogeochemical constraints of each lake, thereby enhancing communication of expected outcomes for bloom control (Erratt et al. 2022).

## Conclusion

The study underscores the complexity of nutrient limitations in cyanobacterial-dominated lakes within agricultural landscapes. Conventional wisdom is challenged by revealing that P and N alone do not fully explain cyanobacterial dominance in the freshwater lakes of the Prairie Pothole Region. The findings from this study highlight an important and often overlooked factor: Fe limitation. In lakes on agricultural landscapes where P and N are abundant, cyanobacterial growth is constrained by the availability of Fe. This Fe limitation forces cyanobacteria to produce siderophores, which may help them acquire the necessary Fe to thrive despite sufficient P and N concentrations. This study highlights the crucial role of Fe in regulating cyanobacterial dynamics, emphasizing its potential for expanding cyanobacterial management. While P and N reductions continue to be central components of lake management, systems with Fe limitations may not experience immediate decreases in biomass from macronutrient reductions, necessitating refined expectations in management. Identifying Fe-limited systems to frame management expectations and exploring iron supplementations could enhance management strategies and improve efforts to control cyanobacterial blooms.

## Supplementary Information

Below is the link to the electronic supplementary material.

Supplementary file1 (PDF 536 KB)

## Data Availability

The data generated in this study are available in the public Zenodo repository: https://doi.org/10.5281/zenodo.13367828.
