# Supplementary material for: Ironing out the question: what is limiting cyanobacteria in freshwater lakes in the Prairie Pothole Region?
Source: Biogeochemistry. 2025 May 20;168(3):51. doi: 10.1007/s10533-025-01234-7 (PMC12092565; doi:10.1007/s10533-025-01234-7)
Supplement: Supplementary file 1 — Supplementary file1 (PDF 536 KB) [file 10533_2025_1234_MOESM1_ESM.pdf]

Supplementary Information

Article Title – Ironing Out the Question: What Is Limiting Cyanobacteria in Freshwater Lakes in the Prairie Pothole Region?

Journal Name – Biogeochemistry

Author Names – Irena F. Creed, Owen Salmon, Kevin J. Erratt, Charles G. Trick

Affiliation – Department of Physical & Environmental Sciences, University of Toronto, Toronto, Ontario, Canada

Corresponding Author – Irena F. Creed

Corresponding Author's Email – irena.creed@utoronto.ca

Table S1. Lake name, identification (ID), geographic coordinates (latitude, longitude), province, and lake and catchment boundary data sources of the study lakes in the Prairie Pothole Region of Canada.

Lake Name	Lake ID	Latitude	Longitude	Province[1]	Lake boundary data source[2]	Catchment boundary data source[3]
Eagle	PPR-001	50.99	-113.32	AB	GPS	HydroSHEDS
Little Fish	PPR-002	51.37	-112.23	AB	GPS	HydroSHEDS
Clear	PPR-003	50.14	-113.41	AB	AGS	SRTM
Park	PPR-004	49.81	-112.93	AB	AGS	HydroSHEDS
Cameron	PPR-006	49.01	-114.05	AB	HydroLAKES	HydroSHEDS
Payne	PPR-007	49.11	-113.65	AB	AGS	HydroSHEDS
Outpost	PPR-008	49.01	-113.46	AB	AGS	HydroSHEDS
Beauvais	PPR-009	49.41	-114.1	AB	GPS	HydroSHEDS
Beaver Mines	PPR-010	49.37	-114.3	AB	AGS	HydroSHEDS
Lee	PPR-011	49.54	-114.25	AB	AGS	HydroSHEDS
Pine	PPR-012	52.08	-113.44	AB	AGS	HydroSHEDS
Chain	PPR-013	52.58	-113.44	AB	AGS	SRTM
Battle	PPR-014	52.97	-114.18	AB	AGS	HydroSHEDS
Halfmoon	PPR-015	53.46	-113.09	AB	AGS	HydroSHEDS
Camp	PPR-016	53.13	-111.53	AB	HydroLAKES	HydroSHEDS
Lac Pelletier	PPR-019	49.98	-107.93	SK	SK-MOE	HydroSHEDS
Elkwater	PPR-021	49.67	-110.29	AB	AGS	HydroSHEDS
Isle	PPR-022	53.63	-114.72	AB	AGS	HydroSHEDS
Lessard	PPR-023	53.79	-114.66	AB	GPS	HydroSHEDS
Thunder	PPR-024	54.13	-114.76	AB	AGS	HydroSHEDS
Lac la Nonne	PPR-025	53.94	-114.33	AB	AGS	HydroSHEDS
Jarvis	PPR-026	53.45	-117.8	AB	AGS	HydroSHEDS
Gregg	PPR-027	53.54	-117.8	AB	AGS	HydroSHEDS
Fickle	PPR-028	53.45	-116.78	AB	AGS	HydroSHEDS

Lake Name	Lake ID	Latitude	Longitude	Province[1]	Lake boundary data source[2]	Catchment boundary data source[3]
Iosegun	PPR-029	54.47	-116.86	AB	AGS	HydroSHEDS
Smoke	PPR-030	54.36	-116.93	AB	AGS	HydroSHEDS
McLeod	PPR-031	54.3	-115.65	AB	AGS	HydroSHEDS
Long	PPR-032	54.42	-112.75	AB	AGS	HydroSHEDS
Skeleton	PPR-033	54.61	-112.72	AB	AGS	HydroSHEDS
Bonnie	PPR-034	54.15	-111.87	AB	AGS	HydroSHEDS
Floatingstone	PPR-035	54.23	-111.63	AB	AGS	HydroSHEDS
Lottie	PPR-036	54.06	-111.6	AB	AGS	HydroSHEDS
Stoney	PPR-037	53.86	-111.09	AB	AGS	HydroSHEDS
Dillberry	PPR-038	52.58	-110.01	AB	AGS	SRTM
Suffern	PPR-039	52.64	-109.9	SK	SK-MOE	HydroSHEDS
Angling	PPR-041	54.2	-110.33	AB	AGS	HydroSHEDS
Minnie	PPR-042	54.28	-111.1	AB	AGS	ALMS
Moose	PPR-043	54.24	-110.91	AB	AGS	HydroSHEDS
Whitney	PPR-044	53.84	-110.55	AB	AGS	HydroSHEDS
Laurier	PPR-045	53.86	-110.51	AB	AGS	HydroSHEDS
Square	PPR-046	54.91	-111.84	AB	AGS	HydroSHEDS
Touchwood	PPR-047	54.82	-111.4	AB	AGS	HydroSHEDS
Elinor	PPR-048	54.65	-111.65	AB	AGS	CDEM
Ironwood	PPR-049	54.6	-111.52	AB	AGS	HydroSHEDS
Crane	PPR-050	54.51	-110.56	AB	AGS	HydroSHEDS
Tucker	PPR-051	54.54	-110.63	AB	AGS	HydroSHEDS
Wolf	PPR-052	53.21	-116.05	AB	HydroLAKES	HydroSHEDS
Kehewin	PPR-053	54.06	-110.9	AB	AGS	HydroSHEDS
Jeannette	PPR-054	54.54	-108.54	SK	SK-MOE	HydroSHEDS
Kimball	PPR-055	54.41	-108.83	SK	SK-MOE	HydroSHEDS
Hirtz	PPR-056	54.48	-109.81	SK	SK-MOE	HydroSHEDS
Fork	PPR-057	54.47	-111.58	AB	AGS	HydroSHEDS
Vincent	PPR-058	54.11	-111.34	AB	AGS	HydroSHEDS
Twins	PPR-059	52.83	-108.52	SK	SK-MOE	HydroSHEDS
Humboldt	PPR-060	52.15	-105.13	SK	SK-MOE	HydroSHEDS
Struther	PPR-062	52.85	-105.24	SK	SK-MOE	HydroSHEDS
Pasqua	PPR-063	50.8	-103.92	SK	SK-MOE	HydroSHEDS
Margo	PPR-065	51.82	-103.36	SK	SK-MOE	HydroSHEDS
Constance	PPR-069	53.17	-106.98	SK	SK-MOE	HydroSHEDS
Jimmy	PPR-070	53.24	-106.87	SK	SK-MOE	HydroSHEDS
Shell	PPR-071	53.22	-107.16	SK	SK-MOE	HydroSHEDS
Brigthsand	PPR-073	53.6	-108.88	SK	GPS	HydroSHEDS
Perch	PPR-074	53.58	-109.47	SK	SK-MOE	HydroSHEDS
Picnic	PPR-075	53.21	-108.67	SK	SK-MOE	HydroSHEDS
Heritage	PPR-077	53.93	-105.16	SK	SK-MOE	HydroSHEDS
Pinkeny	PPR-078	54.05	-105.07	SK	SK-MOE	CDEM
Zeden	PPR-079	53.99	-104.67	SK	SK-MOE	HydroSHEDS
Chante	PPR-082	52.87	-106.11	SK	HydroLAKES	HydroSHEDS

Lake Name	Lake ID	Latitude	Longitude	Province[1]	Lake boundary data source[2]	Catchment boundary data source[3]
York	PPR-087	51.16	-102.48	SK	SK-MOE	HydroSHEDS
Oak	PPR-088	49.67	-100.75	MB	MB-MOECC	HRDEM
Max	PPR-089	49.06	-100.14	MB	MB-MOECC	HydroSHEDS
Bower	PPR-090	49.06	-100.07	MB	MB-MOECC	HydroSHEDS
William	PPR-091	49.04	-99.97	MB	HydroLAKES	HydroSHEDS
Killarney	PPR-092	49.18	-99.68	MB	MB-MOECC	SRTM
Pelican	PPR-093	49.34	-99.57	MB	MB-MOECC	HydroSHEDS
Otter	PPR-095	50.5	-99.85	MB	HydroLAKES	HydroSHEDS
Crawford	PPR-096	50.52	-100.26	MB	HydroLAKES	HydroSHEDS
Sandy	PPR-097	50.53	-100.16	MB	HydroLAKES	HydroSHEDS
Imrie	PPR-098	50.67	-100.22	MB	HydroLAKES	HydroSHEDS

[1] Provinces: AB = Alberta; SK = Saskatchewan; MB = Manitoba.
[2] Lake boundary source: AGS = Alberta Geological Survey (https://ags.aer.ca/products/lake-bathymetry-data); GPS = GPS Nautical Charts (https://www.gpsnauticalcharts.com/main/ca-nautical-charts-by-folio.html); HydroLAKES = HydroLAKES database (version 1.0) (Messager et al. 2016); MB-MOECC = Government of Manitoba Ministry of Environment and Climate Change Wildlife, Fisheries and Resource Enforcement Branch (https://experience.arcgis.com/experience/2557cda82dcc4a348fbb71304cedcf6d/page/Waterbodies/); SK-MOE = Government of Saskatchewan Ministry of the Environment (https://gisappl.saskatchewan.ca/Html5Ext/?viewer=bathy).
[3] Catchment boundary source: ALMS = Alberta Lake Management Society (2023); CDEM = Canadian Digital Elevation Model (https://open.canada.ca/data/en/dataset/7f245e4d-76c2-4caa-951a-45d1d2051333); HydroSHEDS = HydroSHEDS Digital Elevation Model (DEM) (version 1.1) (Lehner et al. 2008); SRTM = Shuttle Radar Topography Mission (https://www.earthdata.nasa.gov/sensors/srtm).

References:

Alberta Lake Management Society (2023) Minnie Lake Report 2022, Updated June 23, 2023. Alberta Lake Management Society Lakewatch Program, Edmonton, https://alms.ca/wp-content/uploads/2023/06/Minnie_2022_FINAL_20230623.pdf. Accessed 10 Aug, 2024

Lehner G, Verdin K, Jarvis A (2008) New global hydrography derived from spaceborne elevation data. Eos 89:93-94. https://doi.org/10.1029/2008EO100001

Messager ML, Lehner B, Grill G, Nedeva I, Schmitt O (2016) Estimating the volume and age of water stored in global lakes using a geo-statistical approach. Nature Comm 7:13603. https://doi.org/10.1038/ncomms1360

Table S2. Lake name, identification (ID), mean Human Impact Index, and physical properties of the study lakes in the Prairie Pothole Region of Canada. Data values that could not be obtained are marked by "NA".

Lake Name	Lake ID	Mean Human Impact Index (0-2)	Catchment land area (km^2)	Percent wetland area in catchment	Percent wetland area connected to lake in catchment	Percent riparian area in catchment	Ratio of catchment land area to lake area	Shoreline length (m)	Shoreline development	Lake fetch (km)	Lake area (ha)	Lake volume (m^3)	Mean lake depth (m)	Maximum lake depth (m)	Percent littoral zone in lake	Dynamic ratio ($km\ m^{-1}$)
Eagle	PPR-001	1.73	129.4	3.03	0.58	0.03	10.8	27,081	2.20	5.86	1,202.1	21,557,951	2.72	5.05	37.06	1.27
Little Fish	PPR-002	1.52	144.8	0.92	0.03	0.12	16.4	14,077	1.34	5.41	880.7	11,236,944	1.38	3.27	71.67	2.15
Clear	PPR-003	1.58	27.3	6.37	2.13	0.04	9.3	13,500	2.22	4.60	293.6	5,148,109	1.75	2.93	52.74	0.98
Park	PPR-004	0.98	0.8	3.71	0.04	10.22	1.0	7,651	2.30	1.91	87.8	1,812,896	2.17	5.95	48.28	0.43
Cameron	PPR-006	0.77	5.3	0.08	0.08	5.65	3.5	6,428	1.48	2.58	149.4	NA	NA	NA	NA	NA
Payne	PPR-007	0.25	23.2	2.67	0.75	1.33	10.6	12,905	2.46	3.50	219.5	5,178,143	2.86	5.92	32.13	0.52
Outpost	PPR-008	0.56	3.4	1.52	1.41	6.05	3.5	5,366	1.53	1.51	97.9	2,236,177	2.94	5.39	32.26	0.34
Beauvais	PPR-009	0.14	7.1	2.00	1.93	4.16	10.9	6,912	2.41	2.61	65.7	490,151	1.20	2.47	87.87	0.68
Beaver Mines	PPR-010	0.14	4.7	0.50	0.50	8.16	6.7	5,341	1.80	1.94	70.1	1,545,070	3.38	5.19	18.53	0.25
Lee	PPR-011	0.46	12.9	0.68	0.24	1.49	34.1	4,961	2.27	1.49	37.9	516,090	2.07	7.20	62.22	0.30
Pine	PPR-012	1.16	160.2	3.50	0.17	0.45	39.6	20,037	2.81	6.76	405.1	NA	NA	NA	22.48	0.40
Chain	PPR-013	0.82	61.2	1.00	0.03	0.65	105.9	6,247	2.32	2.80	57.8	1,709,785	3.38	6.65	27.25	0.22
Battle	PPR-014	0.65	113.6	0.17	0.09	0.95	25.2	18,063	2.40	7.72	450.7	22,849,898	5.96	11.82	11.82	0.36
Halfmoon	PPR-015	0.87	2.8	0.53	0.00	7.37	7.5	4,385	2.03	1.94	37.0	583,760	4.00	6.61	13.53	0.15
Camp	PPR-016	1.65	27.8	4.97	0.00	0.87	38.2	6,554	2.17	2.37	73.0	NA	NA	NA	NA	NA
Lac Pelletier	PPR-019	0.89	65.6	0.19	0.00	0.08	21.8	11,364	1.85	4.91	300.2	10,285,156	3.64	8.89	31.53	0.48
Elkwater	PPR-021	0.41	21.9	0.28	0.04	1.27	9.4	13,220	2.45	3.80	231.4	5,898,282	3.06	8.07	43.27	0.50
Isle	PPR-022	0.92	252.3	0.64	0.22	0.73	11.1	52,618	3.11	14.67	2,277.3	87,014,094	3.95	7.85	22.89	1.21
Lessard	PPR-023	0.72	8.5	0.88	0.88	8.20	2.8	11,047	1.80	2.82	301.2	9,915,436	3.63	6.66	24.99	0.48
Thunder	PPR-024	0.56	21.4	1.37	0.52	3.18	2.8	21,975	2.25	6.10	759.3	21,762,951	2.77	6.37	38.18	0.99
Lac la Nonne	PPR-025	1.10	285.0	0.62	0.04	0.44	22.0	28,462	2.23	7.38	1,294.0	95,512,897	7.35	19.77	21.08	0.49

Lake Name	Lake ID	Mean Human Impact Index (0-2)	Catchment land area (km^2)	Percent wetland area in cathchment	Percent wetland area connected to lake in catchment	Percent riparian area in catchment	Ratio of catchment land area to lake area	Shoreline length (m)	Shoreline development	Lake fetch (km)	Lake area (ha)	Lake volume (m^3)	Mean lake depth (m)	Maximum lake depth (m)	Percent littoral zone in lake	Dynamic ratio (km m^{-1})
Jarvis	PPR-026	0.05	53.0	0.77	0.30	1.82	35.2	15,367	3.54	3.07	150.4	6,929,433	7.04	20.13	17.20	0.17
Gregg	PPR-027	0.11	143.5	0.66	0.16	0.43	104.3	11,312	2.72	3.77	137.6	5,056,189	4.06	15.07	35.81	0.29
Fickle	PPR-028	0.13	112.6	0.55	0.29	0.73	29.4	11,080	1.60	3.58	382.7	8,554,671	2.37	4.74	43.62	0.83
Iosegun	PPR-029	0.61	263.0	1.13	0.19	0.59	19.7	24,394	1.88	8.49	1,338.5	53,626,950	4.22	11.23	35.99	0.87
Smoke	PPR-030	0.31	127.0	0.50	0.11	0.97	13.5	14,873	1.37	5.17	939.6	44,669,062	5.01	8.78	18.57	0.61
McLeod	PPR-031	0.32	49.2	0.47	0.31	0.84	15.6	10,356	1.65	2.63	314.8	13,240,731	5.32	10.72	8.79	0.33
Long	PPR-032	0.26	90.5	0.60	0.10	1.94	14.0	29,889	3.32	11.41	646.3	25,725,374	4.67	9.72	15.32	0.54
Skeleton	PPR-033	0.45	29.4	0.34	0.17	4.85	3.3	24,877	2.37	5.75	878.8	73,071,420	5.68	16.70	23.89	0.52
Bonnie	PPR-034	0.78	46.2	0.45	0.00	2.72	11.1	18,928	2.62	5.34	414.7	10,127,111	2.80	6.31	35.15	0.73
Floatingstone	PPR-035	1.20	245.0	1.47	0.01	0.42	41.0	20,256	2.34	4.12	597.7	31,719,452	5.58	18.70	23.84	0.44
Lottie	PPR-036	1.44	118.1	1.62	0.00	0.55	29.0	18,711	2.61	4.22	407.8	8,272,364	2.16	4.80	51.95	0.93
Stoney	PPR-037	1.17	111.1	2.74	0.00	0.27	47.1	7,895	1.45	2.98	235.8	9,908,380	4.76	9.74	18.45	0.32
Dillberry	PPR-038	0.93	33.6	3.26	0.01	0.00	231.9	2,220	1.65	0.70	14.5	536,114	4.56	9.34	14.65	0.08
Suffern	PPR-039	0.64	15.9	0.43	0.03	0.17	70.2	2,322	1.38	0.91	22.7	538,636	4.02	7.17	10.99	0.12
Angling	PPR-041	0.38	245.1	1.12	0.03	0.13	41.6	9,097	1.06	3.26	588.8	37,029,991	6.77	11.28	11.81	0.36
Minnie	PPR-042	1.37	4.4	1.05	0.00	7.53	5.8	6,059	1.96	1.99	76.2	4,732,205	9.27	19.36	2.20	0.09
Moose	PPR-043	0.41	795.8	1.26	0.05	0.44	19.6	86,611	3.84	13.15	4,052.4	189,561,376	4.44	17.92	39.65	1.43
Whitney	PPR-044	1.04	8.5	1.93	1.38	3.90	6.0	4,504	1.07	1.57	142.0	37,361,463	31.14	42.30	2.36	0.04
Laurier	PPR-045	1.18	133.8	1.78	0.26	0.83	21.7	21,225	2.41	4.82	615.9	23,612,686	4.04	9.14	31.78	0.61
Square	PPR-046	0.13	76.6	3.00	0.32	1.24	12.2	12,829	1.45	3.78	627.5	92,528,752	15.52	37.97	9.52	0.16
Touchwood	PPR-047	0.06	114.7	0.65	0.20	2.34	4.0	38,051	2.00	11.81	2,891.4	405,044,591	14.57	40.81	11.78	0.37
Elinor	PPR-048	0.17	55.7	1.02	0.27	2.70	5.7	28,788	2.61	8.09	971.5	98,598,394	4.89	16.00	27.61	0.64
Ironwood	PPR-049	0.06	142.6	0.86	0.00	0.71	19.1	20,104	2.08	4.59	745.0	79,956,083	12.02	18.87	1.80	0.23
Crane	PPR-050	0.13	49.9	0.61	0.22	2.52	4.9	19,394	1.71	6.68	1,028.9	74,616,381	7.66	26.53	18.20	0.42
Tucker	PPR-051	0.92	257.6	0.75	0.04	0.33	38.7	16,767	1.83	5.63	666.5	14,965,602	2.27	6.71	43.84	1.14

Lake Name	Lake ID	Mean Human Impact Index (0-2)	Catchment land area (km^2)	Percent wetland area in cathchment	Percent wetland area connected to lake in catchment	Percent riparian area in catchment	Ratio of catchment land area to lake area	Shoreline length (m)	Shoreline development	Lake fetch (km)	Lake area (ha)	Lake volume (m^3)	Mean lake depth (m)	Maximum lake depth (m)	Percent littoral zone in lake	Dynamic ratio ($km\ m^{-1}$)
Wolf	PPR-052	0.21	22.0	0.94	0.48	3.51	9.1	10,378	1.88	3.53	242.7	NA	NA	NA	NA	NA
Kehewin	PPR-053	0.75	149.3	0.55	0.01	0.85	22.5	24,875	2.73	9.33	663.1	33,510,191	5.63	11.61	16.17	0.46
Jeannette	PPR-054	0.33	14.3	0.86	0.68	6.06	3.7	13,647	1.96	4.26	387.3	26,526,844	7.82	26.78	7.59	0.25
Kimball	PPR-055	0.16	7.6	3.32	2.05	3.99	2.7	7,656	1.28	2.73	285.6	14,591,256	5.40	13.16	26.15	0.31
Hirtz	PPR-056	0.00	19.3	0.38	0.32	1.28	12.2	5,540	1.24	1.96	158.2	8,958,855	6.44	12.00	10.61	0.20
Fork	PPR-057	0.86	69.8	0.62	0.00	3.16	5.0	40,659	3.07	7.91	1,392.3	76,294,719	5.37	15.45	29.66	0.69
Vincent	PPR-058	1.40	60.7	1.68	0.00	1.52	7.5	20,901	2.07	5.50	815.1	43,003,796	5.48	10.08	20.76	0.52
Twins	PPR-059	1.67	13.7	0.78	0.44	0.24	15.9	4,079	1.24	1.49	86.0	2,881,025	3.15	10.53	41.60	0.29
Humboldt	PPR-060	1.78	183.2	2.34	0.01	0.12	35.0	30,295	3.73	6.99	524.1	18,020,834	2.80	7.44	42.67	0.82
Struther	PPR-062	1.64	905.1	5.14	0.00	0.01	873.9	4,446	1.23	1.64	103.6	3,603,663	4.11	8.32	21.68	0.25
Pasqua	PPR-063	1.71	45,790.7	4.07	0.01	0.00	2,411.6	39,719	2.57	16.54	1,898.8	100,704,174	5.44	15.76	39.63	0.80
Margo	PPR-065	1.76	62.9	1.58	0.01	0.19	24.1	7,108	1.24	2.42	261.0	6,862,308	2.71	6.05	43.06	0.60
Constance	PPR-069	0.87	3.0	5.36	1.87	9.69	5.5	4,913	1.89	2.03	53.7	2,569,805	6.45	18.30	29.49	0.11
Jimmy	PPR-070	0.74	69.1	4.27	0.16	0.30	39.2	9,098	1.93	3.04	176.2	7,120,443	4.48	16.37	35.48	0.30
Shell	PPR-071	0.74	223.9	9.67	0.50	0.15	35.6	17,808	2.00	3.70	628.3	40,938,330	6.73	15.36	21.09	0.37
Brigthsand	PPR-073	1.16	181.5	0.76	0.10	0.61	4.0	27,518	1.16	10.51	4,517.4	241,329,186	5.49	11.24	15.77	1.22
Perch	PPR-074	1.62	24.2	0.89	0.01	0.54	25.0	5,148	1.48	2.14	96.7	3,374,266	5.00	10.89	14.68	0.20
Picnic	PPR-075	1.56	24.2	4.95	0.03	0.50	48.5	2,966	1.18	1.06	50.0	928,096	2.27	5.76	44.99	0.31
Heritage	PPR-077	0.28	10.3	2.91	1.53	5.30	3.7	8,907	1.51	2.75	275.8	16,382,319	6.57	15.25	16.99	0.25
Pinkeny	PPR-078	0.11	49.1	1.87	1.41	0.41	31.8	10,315	2.34	2.16	154.7	4,968,781	3.31	25.62	60.99	0.38
Zeden	PPR-079	0.06	4.7	4.86	0.52	1.04	16.5	2,448	1.29	0.80	28.8	901,348	3.81	8.67	32.03	0.14
Chante	PPR-082	1.62	645.5	5.63	0.00	0.01	989.7	3,957	1.38	1.55	65.2	NA	NA	NA	NA	NA
York	PPR-087	0.89	513.0	6.16	0.00	0.03	147.9	18,688	2.83	7.17	346.9	3,891,895	1.34	3.93	74.24	1.39
Oak	PPR-088	1.24	787.3	3.89	0.13	0.00	29.9	35,887	1.97	8.72	2,630.5	58,881,271	2.21	2.81	22.75	2.32
Max	PPR-089	0.06	18.5	15.44	4.26	5.43	6.9	18,914	3.25	2.96	270.0	5,678,566	3.77	6.01	8.10	0.44

Lake Name	Lake ID	Mean Human Impact Index (0-2)	Catchment land area (km^2)	Percent wetland area in cathcment	Percent wetland area connected to lake in catchment	Percent riparian area in catchment	Ratio of catchment land area to lake area	Shoreline length (m)	Shoreline development	Lake fetch (km)	Lake area (ha)	Lake volume (m^3)	Mean lake depth (m)	Maximum lake depth (m)	Percent littoral zone in lake	Dynamic ratio ($km\ m^{-1}$)
Bower	PPR-090	0.43	2.0	10.15	1.79	13.93	3.5	5,094	1.91	1.17	56.5	1,175,461	3.77	5.62	12.92	0.20
William	PPR-091	0.02	17.9	6.35	0.75	2.34	22.0	6,183	1.93	1.40	81.5	NA	NA	NA	NA	NA
Killarney	PPR-092	1.75	12.8	1.57	0.14	1.23	7.7	16,801	3.68	5.52	166.3	5,087,955	2.80	6.20	41.41	0.46
Pelican	PPR-093	1.66	509.8	1.62	0.04	0.37	18.6	43,115	2.33	17.63	2,733.8	100,214,945	4.10	6.00	8.47	1.28
Otter	PPR-095	0.60	376.7	2.18	0.00	0.14	39.8	23,033	2.11	7.10	946.4	NA	NA	NA	NA	NA
Crawford	PPR-096	1.32	10.1	15.72	0.29	0.11	16.9	4,149	1.51	1.34	59.9	NA	NA	NA	NA	NA
Sandy	PPR-097	1.46	35.6	4.79	0.44	1.01	6.2	22,660	2.67	5.62	573.7	NA	NA	NA	NA	NA
Imrie	PPR-098	0.72	16.9	8.01	0.00	1.30	15.5	7,417	2.01	2.23	108.8	NA	NA	NA	NA	NA

Table S3. Lake name, identification (ID), and chemical properties of the 79 study lakes in the Prairie Pothole Region of Canada: chlorophyll-*a* (Chl-*a*), phycocyanin (PC), microcystin (MC), total phosphorus (TP), and total nitrogen (TN) concentrations, molar N:P ratio, color, Secchi depth, DOC concentration, $SUVA_{254}$, pFe (= -log10[Fe'], where Fe' is the modeled ferric Fe), and siderophore (Sid) concentration in lakes.

Lake Name	Lake ID	Limiting nutrient	Chl-*a* (µg L^{-1})	PC (µg L^{-1})	MC (µg L^{-1})	TP (µg L^{-1})	TN (µg L^{-1})	Molar N:P	Color (TCU)	Secchi depth (m)	DOC (mg L^{-1})	$SUVA_{254}$ (L mg C^{-1} m^{-1})	FI (unitless)	pFe (unitless)	Sid (nmol mL^{-1})
Eagle	PPR-001	Neither	298.6	515.0	169.2	151.3	2,175	14.4	157.9	0.8	26.1	2.6	1.8	24.5	680.7
Little Fish	PPR-002	Neither	369.5	404.3	148.3	442.5	3,050	6.9	98.0	1.0	43.0	1.0	2.1	23.3	752.8
Clear	PPR-003	Neither	113.4	318.4	40.8	140.0	1,590	11.4	17.0	1.1	8.9	0.9	1.7	24.2	501.5
Park	PPR-004	Co-limited	36.0	88.3	6.3	22.8	252	11.1	11.5	3.0	3.2	0.7	2.1	20.2	161.4
Cameron	PPR-006	Co-limited	2.2	0.2	0.4	4.0	90	22.5	2.5	8.1	1.1	1.8	1.6	21.8	102.7
Payne	PPR-007	N-limited	22.9	21.4	12.8	63.5	625	9.8	1.9	2.8	3.2	1.5	1.6	22.6	198.4
Outpost	PPR-008	P-limited	13.8	0.4	0.4	21.0	1,670	79.5	8.6	2.3	20.7	1.2	1.6	24.7	243.6
Beauvais	PPR-009	Co-limited	4.1	0.3	0.4	14.0	476	34.0	3.4	3.3	6.5	2.7	1.6	22.6	186.2
Beaver Mines	PPR-010	Co-limited	53.6	3.9	10.9	22.3	370	16.6	7.3	3.4	3.4	1.9	1.6	22.9	160.8
Lee	PPR-011	P-limited	9.2	0.3	0.4	12.0	804	67.0	7.1	2.2	9.0	1.4	2.0	24.4	195.8
Pine	PPR-012	Neither	201.7	628.4	247.3	290.0	2,965	10.2	45.5	1.2	22.5	1.7	1.8	24.6	506.1
Chain	PPR-013	Neither	132.5	397.8	64.8	73.0	1,193	16.3	50.8	0.5	17.6	2.2	1.8	23.5	599.9
Battle	PPR-014	Co-limited	168.7	387.6	20.9	28.3	498	17.6	22.9	0.7	11.0	2.4	1.7	24.8	533.1
Halfmoon	PPR-015	Neither	214.6	239.9	68.8	52.8	1,515	28.7	33.8	0.8	24.0	1.4	1.9	24.6	526.5
Camp	PPR-016	Neither	83.4	197.8	105.7	70.0	1,615	23.1	27.1	0.3	47.9	1.0	1.6	25.4	550.8
Lac Pelletier	PPR-019	Neither	69.5	126.5	43.3	111.5	1,253	11.2	14.3	0.8	10.7	1.6	1.8	24.7	799.9
Elkwater	PPR-021	Co-limited	29.0	121.1	0.4	13.3	535	40.4	13.2	1.8	9.0	1.8	1.7	22.8	359.8
Isle	PPR-022	Neither	344.2	505.7	185.5	277.5	2,525	9.1	34.0	0.7	22.0	1.7	1.8	22.4	625.3
Lessard	PPR-023	Neither	35.9	28.9	1.4	69.0	2,190	31.7	27.3	0.8	22.4	1.6	1.8	20.5	313.5
Thunder	PPR-024	Neither	98.0	298.3	148.7	53.0	2,320	43.8	24.1	0.6	25.7	1.4	1.7	25.1	512.8
Lac la Nonne	PPR-025	Neither	148.5	264.5	18.7	290.0	2,240	7.7	23.1	1.1	18.1	1.6	1.7	25.3	527.9
Jarvis	PPR-026	Co-limited	5.2	0.2	0.4	14.0	307	21.9	21.4	3.1	17.6	0.9	1.6	23.8	203.7
Gregg	PPR-027	Co-limited	5.2	0.2	0.4	10.0	340	34.0	33.1	3.5	15.2	1.4	1.6	20.9	237.7

**Lake Name**	**Lake ID**	**Limiting nutrient**	**Chl-*a* ($\mu g\ L^{-1}$)**	**PC ($\mu g\ L^{-1}$)**	**MC ($\mu g\ L^{-1}$)**	**TP ($\mu g\ L^{-1}$)**	**TN ($\mu g\ L^{-1}$)**	**Molar N:P**	**Color (TCU)**	**Secchi depth (m)**	**DOC ($mg\ L^{-1}$)**	**$SUVA_{254}$ ($L\ mg\ C^{-1}\ m^{-1}$)**	**FI (unitless)**	**pFe (unitless)**	**Sid ($nmol\ mL^{-1}$)**
Fickle	PPR-028	Neither	74.2	154.9	5.0	59.0	929	15.7	88.3	1.3	19.9	2.9	1.7	19.1	513.0
Iosegun	PPR-029	Neither	154.8	345.2	36.3	113.0	1,130	10.0	146.4	0.7	24.2	3.2	1.6	18.3	561.8
Smoke	PPR-030	Neither	101.5	269.4	21.4	82.8	813	9.8	93.1	1.6	19.0	3.1	1.6	19.2	766.9
McLeod	PPR-031	Co-limited	10.9	29.0	6.2	12.5	199	15.9	21.8	2.6	14.1	1.7	1.6	23.3	236.4
Long	PPR-032	N-limited	56.4	428.0	144.0	60.8	798	13.1	28.3	0.3	17.5	1.7	1.7	24.3	716.7
Skeleton	PPR-033	Neither	88.6	112.2	104.8	268.0	3,460	12.9	23.3	0.2	22.4	1.3	1.7	25.2	676.7
Bonnie	PPR-034	Neither	306.9	472.3	485.3	315.0	4,938	15.7	40.9	0.6	41.0	1.3	2.0	23.1	457.0
Floatingstone	PPR-035	Co-limited	79.0	149.8	19.8	36.3	793	21.9	19.3	0.3	19.6	1.2	1.7	25.6	675.0
Lottie	PPR-036	Neither	118.5	552.0	49.0	267.5	3,050	11.4	52.0	0.4	38.5	1.6	1.8	24.5	558.2
Stoney	PPR-037	Neither	111.9	107.3	8.7	78.0	2,110	27.1	23.1	0.8	22.7	1.5	1.8	24.9	476.9
Dillberry	PPR-038	P-limited	9.3	0.4	0.4	18.0	809	44.9	8.0	3.6	10.8	1.2	1.7	23.8	87.5
Suffern	PPR-039	P-limited	10.6	4.3	0.4	16.0	899	56.2	8.2	2.7	13.2	1.1	1.7	24.9	234.8
Angling	PPR-041	Co-limited	138.2	190.6	3.4	41.5	800	19.3	34.8	0.8	19.4	1.2	1.9	25.6	541.0
Minnie	PPR-042	P-limited	17.1	2.7	0.4	27.0	1,390	51.5	6.7	2.9	21.3	0.8	1.8	24.9	223.6
Moose	PPR-043	Neither	85.7	133.4	0.4	108.0	1,355	12.5	26.0	0.7	20.5	1.4	1.8	24.7	655.2
Whitney	PPR-044	P-limited	7.6	0.4	0.4	15.0	1,330	88.7	15.5	3.1	22.2	1.3	1.7	24.8	241.6
Laurier	PPR-045	Neither	65.9	52.2	3.3	136.5	1,555	11.4	28.5	0.8	33.8	1.3	1.8	25.4	205.0
Square	PPR-046	P-limited	82.1	88.5	2.1	43.5	898	20.6	26.0	0.6	22.6	1.3	1.9	25.5	567.7
Touchwood	PPR-047	Co-limited	5.9	0.4	0.4	13.0	510	39.2	8.0	4.1	13.6	1.1	1.6	24.7	222.7
Elinor	PPR-048	N-limited	83.3	16.3	39.0	56.0	770	13.8	24.1	0.7	26.0	1.1	1.8	24.9	545.8
Ironwood	PPR-049	P-limited	14.7	0.4	0.4	25.0	1,200	48.0	15.9	3.1	24.2	1.0	1.7	26.0	239.1
Crane	PPR-050	P-limited	6.0	0.8	0.4	22.0	875	39.8	6.1	4.2	19.1	0.7	1.7	25.2	315.0
Tucker	PPR-051	Neither	85.0	252.7	36.3	66.0	1,190	18.0	21.2	0.8	14.5	1.8	1.7	23.9	509.1
Wolf	PPR-052	P-limited	20.7	36.1	0.4	23.0	1,050	45.7	15.1	3.4	15.7	1.5	1.6	23.6	218.6
Kehewin	PPR-053	P-limited	108.5	228.3	4.6	48.5	868	17.9	23.5	0.3	15.7	1.5	1.8	25.2	598.6
Jeannette	PPR-054	Co-limited	5.4	0.4	0.4	5.0	450	90.0	2.7	5.6	7.9	0.7	1.7	24.3	194.4
Kimball	PPR-055	Co-limited	3.5	1.1	0.4	11.0	629	57.2	2.7	4.6	12.1	0.8	1.6	24.7	158.1
Hirtz	PPR-056	P-limited	50.5	113.8	5.3	45.0	915	20.3	18.7	2.3	16.6	1.4	1.8	25.3	582.9
Fork	PPR-057	P-limited	62.9	137.8	87.8	46.0	1,800	39.1	10.7	0.6	21.8	1.0	1.8	25.3	475.4

Lake Name	Lake ID	Limiting nutrient	Chl-*a* (µg L^{-1})	PC (µg L^{-1})	MC (µg L^{-1})	TP (µg L^{-1})	TN (µg L^{-1})	Molar N:P	Color (TCU)	Secchi depth (m)	DOC (mg L^{-1})	$SUVA_{254}$ (L mg C^{-1} m^{-1})	FI (unitless)	pFe (unitless)	Sid (nmol mL^{-1})
Vincent	PPR-058	Co-limited	56.3	189.5	8.3	20.0	748	37.4	46.4	0.5	31.9	1.2	2.0	25.0	450.0
Twins	PPR-059	Co-limited	68.3	106.0	57.8	31.8	795	25.0	23.5	0.3	17.0	1.4	1.7	24.9	575.8
Humboldt	PPR-060	Neither	67.5	317.5	93.0	77.8	993	12.8	40.7	0.6	22.1	1.6	1.8	23.2	776.8
Struther	PPR-062	Co-limited	59.1	114.7	3.3	14.5	708	48.8	45.3	0.5	20.7	2.1	1.7	23.5	525.7
Pasqua	PPR-063	Co-limited	47.2	117.9	2.8	35.3	463	13.1	31.9	0.7	14.7	1.7	1.8	24.0	337.0
Margo	PPR-065	Neither	143.2	327.8	11.1	82.0	2,300	28.0	31.7	0.6	17.3	1.9	1.8	24.0	669.9
Constance	PPR-069	P-limited	4.0	0.5	0.4	10.0	1,240	124.0	10.1	4.1	18.8	1.2	1.7	25.3	311.8
Jimmy	PPR-070	P-limited	7.5	1.6	0.4	18.0	1,250	69.4	17.6	3.9	19.4	1.5	1.7	24.7	298.4
Shell	PPR-071	Neither	16.8	1.0	8.4	64.0	1,280	20.0	23.5	2.9	11.6	1.7	1.7	25.0	232.1
Brigthsand	PPR-073	P-limited	7.9	0.5	0.4	10.0	872	87.2	2.7	4.1	13.8	0.5	1.7	24.6	87.8
Perch	PPR-074	Neither	91.4	148.0	37.3	149.0	3,480	23.4	24.3	0.3	20.4	1.6	1.6	25.1	726.8
Picnic	PPR-075	P-limited	8.1	14.7	0.4	15.0	1,120	74.7	12.2	2.9	12.6	1.5	1.7	24.9	211.9
Heritage	PPR-077	Co-limited	3.7	1.9	0.4	13.0	602	46.3	6.5	4.1	10.2	1.3	1.7	24.4	215.8
Pinkeny	PPR-078	Co-limited	9.0	0.4	0.4	25.0	647	25.9	17.4	3.8	11.1	1.7	1.6	21.2	179.7
Zeden	PPR-079	Co-limited	4.0	29.5	0.4	8.0	424	53.0	4.2	4.2	6.7	1.2	1.7	23.3	178.2
Chante	PPR-082	P-limited	53.2	170.6	32.4	29.0	1,180	40.7	18.9	0.3	32.7	1.3	1.7	25.1	671.3
York	PPR-087	Neither	88.1	205.1	63.7	90.0	1,388	15.4	27.3	0.6	33.6	1.2	1.8	23.7	523.6
Oak	PPR-088	Neither	107.2	381.4	30.3	89.0	1,283	14.4	70.9	0.7	20.2	2.0	1.8	21.6	534.1
Max	PPR-089	P-limited	20.9	39.0	2.3	40.0	1,320	33.0	14.7	2.7	13.9	1.4	1.7	24.5	229.3
Bower	PPR-090	Co-limited	36.2	51.1	0.4	22.5	500	22.2	18.5	0.7	12.8	1.3	1.7	24.1	308.9
William	PPR-091	P-limited	8.2	0.5	0.4	15.0	1,010	67.3	13.2	2.4	14.1	1.4	1.7	24.2	280.7
Killarney	PPR-092	Neither	233.5	478.4	79.0	120.5	1,613	13.4	35.7	0.5	18.7	1.5	1.9	23.9	495.6
Pelican	PPR-093	Neither	119.0	321.1	178.6	257.5	1,065	4.1	26.4	0.6	15.3	1.4	1.8	24.5	587.5
Otter	PPR-095	Neither	81.6	258.4	67.8	107.3	870	8.1	33.6	0.4	18.7	1.7	1.7	25.1	609.0
Crawford	PPR-096	Co-limited	40.8	59.0	6.7	27.0	700	25.9	19.1	0.9	21.0	1.4	1.8	24.6	273.1
Sandy	PPR-097	Neither	58.3	112.2	8.4	64.0	2,550	39.8	25.2	0.8	25.4	1.2	1.8	24.3	228.2
Imrie	PPR-098	P-limited	5.2	1.8	0.4	13.0	1,030	79.2	16.8	3.2	13.0	1.6	1.8	23.4	174.7

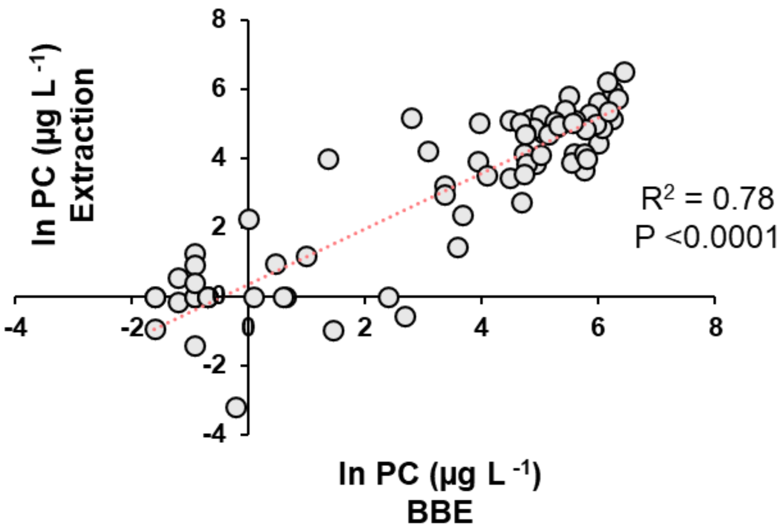

Figure S1 – Relationship between extracted phycocyanin and fluorometrically determined in-situ phycocyanin concentrations in lakes. BBE refers to bbe moldaenke, which is the instrument that uses fluorescence measurements to quantify chlorophyll concentration and identify distinct algae groups based on their unique fluorescence "fingerprints."

Table S4. Lake name, identification (ID), and chemical properties of the 79 study lakes in the Prairie Pothole Region of Canada, including total chlorophyll-a (Chl-a), blue-green algae (phycocyanin, PC), and the percentage of blue-green algae, as measured by the BBE PhycoLab Analyzer.

**Lake Name**	**Lake ID**	**Total Chl-*a* (µg L^{-1})**	**Blue-green algae (PC) (µg L^{-1})**	**Blue-green algae (PC) (%)**
Eagle	PPR-001	213.95	167.59	78.3
Little Fish	PPR-002	133.03	83.26	62.6
Clear	PPR-003	45.02	38.39	94.6

Lake Name	Lake ID	Total Chl-*a* (µg L^{-1})	Blue-green algae (PC) (µg L^{-1})	Blue-green algae (PC) (%)
Park	PPR-004	61.89	30.66	55.5
Cameron	PPR-006	2.75	0.00	0.0
Payne	PPR-007	102.93	67.91	66.0
Outpost	PPR-008	17.48	3.49	30.8
Beauvais	PPR-009	6.26	0.84	26.8
Beaver Mines	PPR-010	80.32	54.12	84.3
Lee	PPR-011	8.96	1.73	36.8
Pine	PPR-012	753.44	666.37	98.1
Chain	PPR-013	298.00	272.52	95.2
Battle	PPR-014	161.88	144.29	92.6
Halfmoon	PPR-015	373.72	331.48	93.8
Camp	PPR-016	207.98	136.13	70.7
Lac Pelletier	PPR-019	237.08	171.10	90.9
Elkwater	PPR-021	72.65	56.09	80.8
Isle	PPR-022	445.01	381.06	94.4
Lessard	PPR-023	67.29	24.37	39.9
Thunder	PPR-024	86.55	58.04	86.3
Lac la Nonne	PPR-025	79.59	62.81	88.7
Jarvis	PPR-026	7.45	0.00	5.5
Gregg	PPR-027	7.23	0.39	20.9
Fickle	PPR-028	173.53	112.31	90.7
Iosegun	PPR-029	220.40	193.38	96.6
Smoke	PPR-030	183.16	161.03	93.8
McLeod	PPR-031	33.72	18.81	64.7
Long	PPR-032	166.12	129.40	84.3
Skeleton	PPR-033	94.53	63.25	82.7
Bonnie	PPR-034	584.68	486.50	93.5
Floatingstone	PPR-035	249.72	188.97	84.6
Lottie	PPR-036	378.82	304.09	88.5

Lake Name	Lake ID	Total Chl-*a* (µg L^{-1})	Blue-green algae (PC) (µg L^{-1})	Blue-green algae (PC) (%)
Stoney	PPR-037	24.55	15.14	71.6
Dillberry	PPR-038	9.41	0.01	12.4
Suffern	PPR-039	9.70	0.38	37.2
Angling	PPR-041	189.24	135.90	81.6
Minnie	PPR-042	12.52	3.18	43.0
Moose	PPR-043	198.08	129.07	76.0
Whitney	PPR-044	7.82	0.00	6.9
Laurier	PPR-045	234.09	150.87	90.0
Square	PPR-046	394.83	160.70	49.4
Touchwood	PPR-047	10.78	0.24	13.6
Elinor	PPR-048	280.02	177.03	75.7
Ironwood	PPR-049	17.50	2.48	22.6
Crane	PPR-050	7.44	0.04	25.7
Tucker	PPR-051	61.23	47.24	82.2
Wolf	PPR-052	12.33	4.18	48.0
Kehewin	PPR-053	291.65	222.52	84.9
Jeannette	PPR-054	4.09	0.00	17.4
Kimball	PPR-055	4.95	0.00	19.8
Hirtz	PPR-056	127.10	113.40	89.2
Fork	PPR-057	66.57	45.62	78.6
Vincent	PPR-058	197.88	155.27	84.7
Twins	PPR-059	217.99	148.67	89.1
Humboldt	PPR-060	87.76	61.49	78.0
Struther	PPR-062	126.92	106.80	87.8
Pasqua	PPR-063	106.43	46.51	51.9
Margo	PPR-065	82.06	53.07	84.0
Constance	PPR-069	5.79	0.00	9.8
Jimmy	PPR-070	9.95	2.54	33.3
Shell	PPR-071	24.96	9.44	50.0

Lake Name	Lake ID	Total Chl-*a* (µg L^{-1})	Blue-green algae (PC) (µg L^{-1})	Blue-green algae (PC) (%)
Brigthsand	PPR-073	11.02	0.00	18.2
Perch	PPR-074	149.73	59.15	55.4
Picnic	PPR-075	12.29	0.56	16.2
Heritage	PPR-077	6.04	0.00	12.6
Pinkeny	PPR-078	11.02	1.45	21.6
Zeden	PPR-079	6.93	0.00	0.0
Chante	PPR-082	176.99	106.48	79.7
York	PPR-087	300.48	141.19	66.0
Oak	PPR-088	181.28	146.73	91.9
Max	PPR-089	17.07	10.43	68.2
Bower	PPR-090	116.16	49.70	68.6
William	PPR-091	12.00	0.00	24.8
Killarney	PPR-092	251.92	210.40	93.4
Pelican	PPR-093	170.44	126.19	84.3
Otter	PPR-095	224.48	150.10	75.1
Crawford	PPR-096	103.66	33.41	39.8
Sandy	PPR-097	57.88	34.06	74.1
Imrie	PPR-098	7.35	0.00	10.9
